# Electronic Properties of Functionalized Diamanes for Field-Emission Displays

**DOI:** 10.1021/acsami.3c01536

**Published:** 2023-03-16

**Authors:** Christian Tantardini, Alexander G. Kvashnin, Maryam Azizi, Xavier Gonze, Carlo Gatti, Tariq Altalhi, Boris I. Yakobson

**Affiliations:** † Hylleraas Center, Department of Chemistry, UiT The Arctic University of Norway, P.O. Box 6050 Langnes, N-9037 Tromsø, Norway; ‡ Department of Materials Science and NanoEngineering, Rice University, Houston, Texas 77005, United States; § Institute of Solid State Chemistry and Mechanochemistry SB RAS, Novosibirsk 630128, Russian Federation; ∥ Skolkovo Institute of Science and Technology, Bolshoi Boulevard 30, Building 1, Moscow 121205, Russian Federation; ⊥ Université catholique de Louvain, Place de l’Université 1, Ottignies-Louvain-la-Neuve 1348, Belgium; # SCITEC - Istituto di Scienze e Tecnologie Chimiche “Giulio Natta”, CNR - Consiglio Nazionale delle Ricerche, sezione di via Golgi, 19, Milan 20133, Italy; ∇ Chemistry Department, Taif University, Al Hawiyah, Taif 26571, Saudi Arabia

**Keywords:** diamanes, ABINIT, meta-GGA, *GW* approximation, Bader theory, Tantardini−Oganov electronegativity, field-emission displays

## Abstract

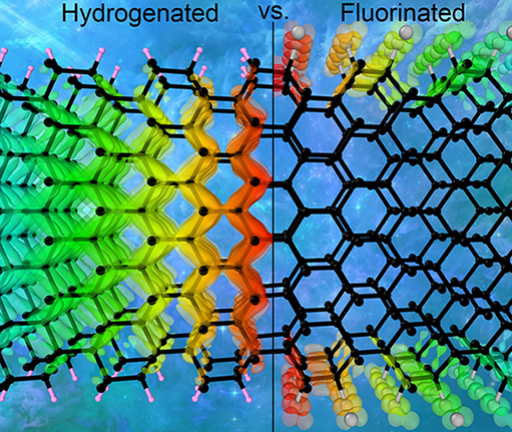

Ultrathin diamond films, or diamanes, are promising quasi-2D materials that are characterized by high stiffness, extreme wear resistance, high thermal conductivity, and chemical stability. Surface functionalization of multilayer graphene with different stackings of layers could be an interesting opportunity to induce proper electronic properties into diamanes. Combination of these electronic properties together with extraordinary mechanical ones will lead to their applications as field-emission displays substituting original devices with light-emitting diodes or organic light-emitting diodes. In the present study, we focus on the electronic properties of fluorinated and hydrogenated diamanes with (111), (110), (0001), (101̅0), and (2̅110) crystallographic orientations of surfaces of various thicknesses by using first-principles calculations and Bader analysis of electron density. We see that fluorine induces an occupied surface electronic state, while hydrogen modifies the occupied bulk state and also induces unoccupied surface states. Furthermore, a lower number of layers is necessary for hydrogenated diamanes to achieve the convergence of the work function in comparison with fluorinated diamanes, with the exception of fluorinated (110) and (2̅110) films that achieve rapid convergence and have the same behavior as other hydrogenated surfaces. This induces a modification of the work function with an increase of the number of layers that makes hydrogenated (2̅110) diamanes the most suitable surface for field-emission displays, better than the fluorinated counterparts. In addition, a quasi-quantitative descriptor of surface dipole moment based on the Tantardini–Oganov electronegativity scale is introduced as the average of bond dipole moments between the surface atoms. This new fundamental descriptor is capable of predicting a priori the bond dipole moment and may be considered as a new useful feature for crystal structure prediction based on artificial intelligence.

## Introduction

Nowadays, the possibilities of applications of two-dimensional materials for electronic devices are scrutinized by numerous research groups worldwide. The search for new materials for application in field-emission displays (FEDs) represents a very hot topic due to the necessity to produce flatter panels (i.e., approximately 2 mm) with the characteristics of self-emissive distortion-free images and wide view angles (i.e., about 170°). Furthermore, FEDs are characterized by quick response, in the order of microseconds, tolerance to environments as high as that of receiving tubes, and free from terrestrial and applied magnetic effects.^1−6^ These characteristics make FEDs more appreciated than corresponding light-emitting diodes (LEDs), organic light-emitting diodes (OLEDs), and surface-conduction electron-emitter displays (SEDs).^1−6^ The search for new materials employed as FEDs benefits enormously from a deep study of the electronic structure at the surface, which in most cases is peculiar and differs from the bulk, due to the significantly changed chemistry caused by the surface-modified bonding pattern.

Considering carbon materials, one notes that diamond and lonsdaleite are both sp^3^-hybridized insulating allotropes, while their 2D counterpart graphene^7,8^ is a semimetal with sp^2^-hybridized carbon atoms. Indeed, the hybridization plays an important role not only in the chemistry but also in the electronic properties of carbon materials. In particular, the surface functionalization of multilayer graphene with different stackings of layers by different atoms enables chemically induced phase transition converting multilayer graphene into diamond-type structures with sp^3^-hybridized carbon atoms in all layers leading to semiconducting properties.^9−17^ These functionalized multilayers are different from graphene that presents sp^2^ hybridization of carbons and electronic properties of a semimetal. The sp^3^-hybridized multilayers are quasi-2D compounds called diamanes, various structures of which are caused by AA, AA′, or AB stackings of graphene layers.^9,11−17^ More exotic structures can be formed by fabrication of moiré patterns from bilayer graphene functionalized by hydrogen or fluorine,^16,18−22^ and even quasicrystals can be formed.^23^

Indeed, the formation of diamanes from multilayer graphene via the application of low temperature and pressure (∼50 Torr) was observed experimentally in refs.^24,25^ Authors applied the hot-filament process for the efficient hydrogenation and dehydrogenation of few-layered graphene and the subsequent formation of crystalline and ultrathin sp^3^ carbon sheets observed.

Diamanes exhibit a unique combination of physical properties such as high thermal conductivity,^26−31^ which is compatible with small-polaron charge carriers, and optical characteristics,^32−34^ making them suitable for potential applications in electromechanical devices.^30^ In addition, diamanes with different functionalizations of surfaces could be good candidates for FEDs. Indeed, 2D materials are also remarkable due to the possibility of doping their surface in a reversible way by functionalization/defunctionalization. Hence, the switching of electronic properties from metals to semiconductors and vice versa occurs due to the chemical or photochemical reactions. A sort of reversible photodoping was recently discovered by Gierster et al.^35^ at the (101̅0) surface of ZnO, where phase transition is caused by photoinduced downward surface band bending due to photodepletion of donor-type deep surface defects.

Hydrogenated diamanes are a case of reversible chemical doping.^24,36−39^ Furthermore, reversible fluorination of few-layer graphene was also experimentally achieved in refs.^40−43^ Actually, for both hydrogenated and fluorinated diamanes, the energy barrier of hydrogenation or fluorination decreases with the number of layers and the monolayer is the most difficult structure to be formed from this point of view.^13,14,44^

Thus, to understand the possibility of using diamanes in FEDs, a state-of-the-art fundamental study of their electronic properties with first-principles calculations is much needed, which is the goal of the present work. Indeed, the Kohn–Sham (KS) electronic band structure together with *GW* approximation gap values^45^ allow us to understand the dependence of the work function on the functionalization type and thickness of diamanes. A subsequent study of electron population at the valence band maxima (VBM) within the framework of Bader theory^46,47^ applied for 2D materials^48,49^ enables us to understand the atomic contribution responsible for the conductivity, making clear the electronic transport behavior at the atomic level in view of the future development of 2D optoelectronic devices based on diamanes.

## Results and Discussion

**Figure 1:**
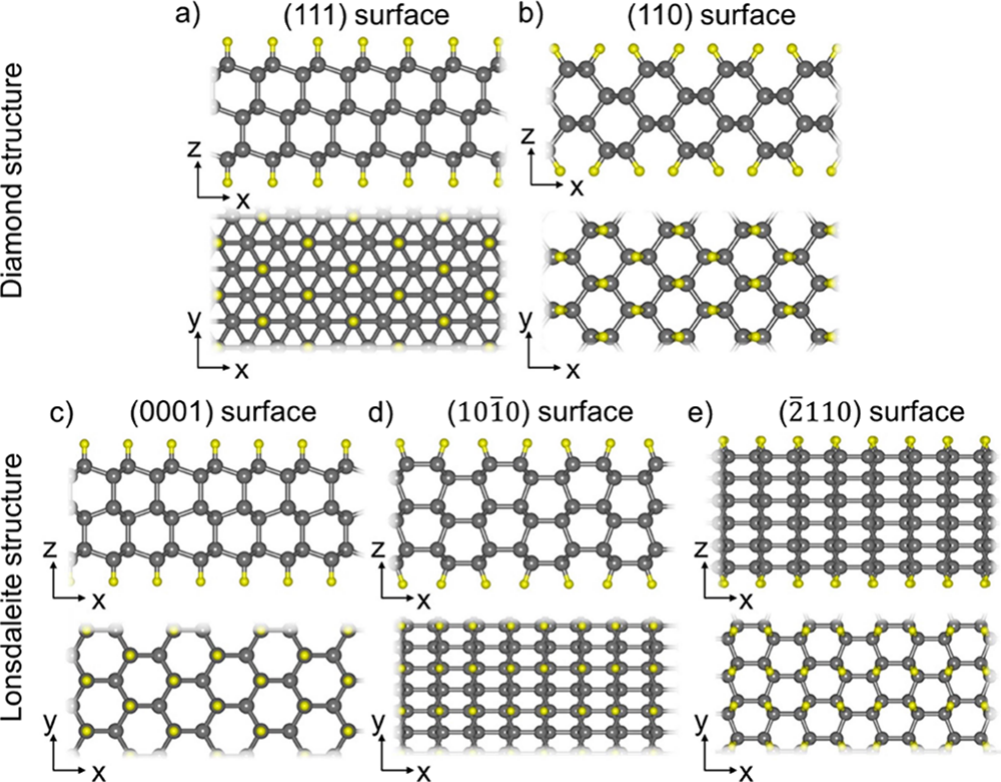
Top and side views of atomic structures of the considered diamanes with diamond and lonsdaleite structures having (a) (111), (b) (110), (c) (0001), (d) (101̅0), and (e) (2̅110) surfaces respectively. Gray balls represent carbon atoms and yellow balls are noncarbon atoms (H or F).

Five types of diamanes are considered, having (111), (110), (0001), (101̅0), and (2̅110) crystallographic orientations of surfaces, with thickness from 1 to 6 layers and hydrogen or fluorine functionalization, so altogether 60 structures. Hydrogen and fluorine atoms are bonded with the surface carbon atoms through a covalent bonds. Thus, all carbon atoms in the considered diamanes are sp^3^-hybridized. Atomic structures of these five types of diamanes are shown in Figure 1. Diamanes with (111) and (110) surfaces belong to a group of films with the cubic diamond structure type, while other films have the lonsdaleite (hexagonal diamond) structure type. Yellow atoms in Figure 1 represent noncarbon atoms (hydrogen or fluorine, which are presented here). Films with (0001) and (2̅110) surfaces can be formed by passivation of graphene multilayers with AA stacking, while AA’ stacking leads to formation of (101̅0) films.^12^

For each considered diamane, the KS electronic band structure is calculated by using the TB09 (aka modified Becke–Johnson, a meta-generalized gradient approximation (GGA) functional) DFT exchange-correlation functional^50^ on top of a GGA-PBE optimized structure, as shown in Figures S1–S10 in the Supporting Information. The TB09 functional is quite efficient in calculating the accurate band gap of various bulk materials, with respect to LDA and GGA-PBE, and is in a reasonable agreement with the *GW* approximation or experimental data for bulk solids.^50−52^ This makes it a priori an excellent starting point to achieve useful information about electronic band structures and band gaps for 3D structures^53^ and we will test it here on 2D structures.

All hydrogenated (111), (110), (0001), (101̅0), and (2̅110) diamanes possess direct TB09 KS band gaps (Figures S1–S10 in the Supporting Information). Few-layer fluorinated (111), (110), (0001), and (101̅0) diamanes are characterized by direct band gaps, while an increase in thickness leads to the appearance of an indirect band gap, with the exception of (2̅110), which is a direct gap semiconductor for all considered thicknesses (Figures S1–S10 in the Supporting Information). As we know, the band gap analysis gives a crucial information about the system. However, the accuracy of the KS electronic band structure is affected by the DFT problem that underestimates the band gap and overestimates the electronic repulsion with the Hartree term that is not always properly balanced with the exchange-correlation functional.

**Figure 2:**
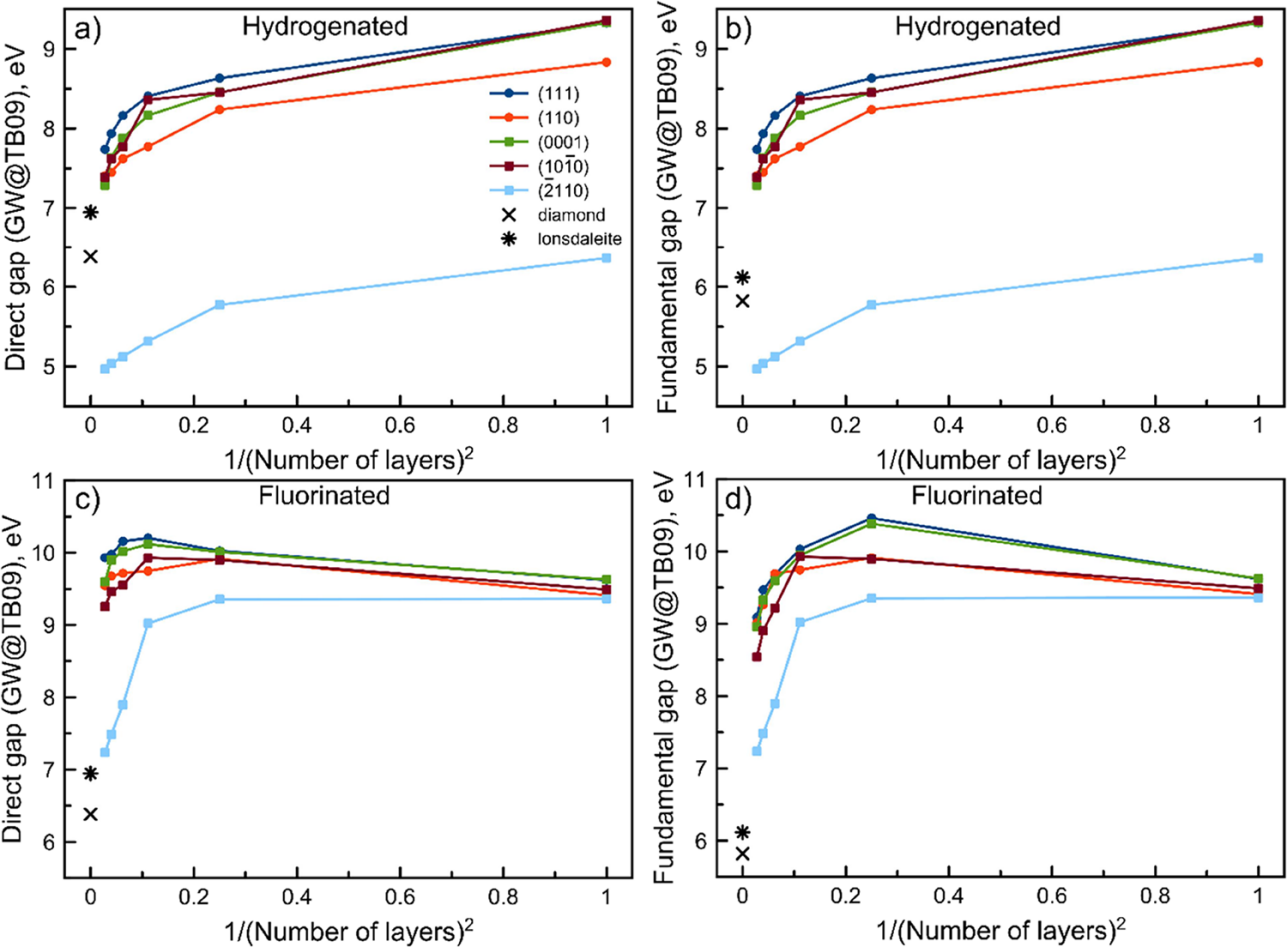
Dependency on the squared inverse number of layers (thickness) of the direct (a, c) and fundamental (b, d) band gaps for hydrogenated (a, b) and fluorinated (c, d) diamanes, calculated by the *GW@*TB09 approach.

To evaluate the quantitative prediction of band gaps in 2D solids obtained with TB09, we have subsequently performed *G*_0_*W*_0_ calculations on top of the TB09 DFT results (from now on, referred to as *GW*@TB09). Furthermore, we have compared fundamental and direct gaps obtained with *GW*@TB09 as a function of the inverse squared number of layers (see Figures 2 and S11 in the Supporting Information). This was done according to the fact that in quantum mechanics, the energy level spacing of a particle in a square box (like a confined electron) is a function of the inverse squared box size. Theoretically, an increase in the number of layers (*L*) of diamanes should lead to a decrease in the band gap until converging to the value for bulk lonsdaleite or diamond in the limit of *L* →∞. In reality, such behavior is preserved for bulk states, but modifications do not follow the same law for surface state(s).

Moreover, comparing the *GW*@TB09 (see Figure 2) with TB09 (see Figure S11 in the Supporting Information) band gap dependencies, we observe two totally different behaviors. Both fundamental and direct band gaps of all hydrogenated diamanes calculated by TB09 decrease with the increase of the number of layers and starting from three layers, band gaps start to increase, Figure S11a,b. Fluorinated films demonstrate nonmonotonic behavior of band gaps with thickness (Figure S11c,d). Interestingly, this band gap behavior of (2̅110) diamanes is counterintuitive, namely, there is no tendency to the band gaps of bulk lonsdaleite with *L* →∞. More accurate *GW*@TB09 corrects this situation, as shown in Figure 2. In order to double-check our *GW*@TB09 predictions, we examined whether there might be a reordering of electronic bands calculated by TB09 and *G*_0_*W*_0_ that might change the electronic states in hydrogenated (111), (110), (0001), (101̅0), and (2̅110) diamanes at the band gap extrema. We have monitored the energy difference between the bands at VBM with respect to the closest highest bands, confirming that no reordering occurs. Thus, while TB09 is considered as one of the best choices for the KS band structure for 3D materials,^53^ for 2D systems, its predictive capability seems degraded.

From our *GW*@TB09 results (see Figure 2), the increase of the number of layers in hydrogenated diamanes always induces a decrease of the band gap from its initial value (monolayer, graphane). At variance, in fluorinated diamanes, the band gap is seen to initially increase with the number of layers from the monolayer and subsequently decrease after three layers, to converge, in the larger thickness, to the corresponding band gap of the bulk counterpart. Furthermore, only hydrogenated (2̅110) films display a clear band gap behavior that does not converge to the band gap of lonsdaleite with the increase of the number of layers (blue curves in Figure 2).

The band gap behavior is affected by different functionalizations of the surfaces, which are correlated with the electronic states. This can be understood by looking at the KS electronic band structures (Figures S1–S10 in the Supporting Information) that are not affected by reordering, as was checked previously with *GW* calculations. KS TB09 electronic band structures (see in Supporting Information Figures S1–S10) are intricate and benefit from further investigation using Bader theory.

Bulk electronic states in such multilayers are characterized by the Bloch character of the wavefunction in the inner layers while decaying exponentially into a vacuum. Instead, surface electronic states are characterized by exponential decay both in vacuum and in the inner layers and thus represents states localized at the crystal surface.

These two types of states, from the occupied bulk valence states and from the occupied surface states, contribute to the charge density of the bulk and surface with different atomic characteristics.

Actually, in the presence of a few layers, it is difficult to speak about localized surface electronic states, because the whole few-layer structure is made of the surface. Thus, the localization can come only in the presence of thicker multilayers. Thus, we can suppose that the different band gap behaviors for fluorinated and hydrogenated diamanes with the increase of layers are due to the formation of surface electronic states in one case and due to bulk electronic states in the other.

Two different electronic states can be recognized by different electronic atomic contribution to the VBM: an electronic atomic contribution coming from all bulk atoms is responsible for a bulk state; an electronic contribution coming for specific localized atoms is responsible for a surface electronic state. This can be quantified through a Bader analysis.

In the case of surface electronic states, the charge density at the VBM might come from surface adatoms, as well as from the carbon atoms adjacent to these. The latter is responsible for the variation of electronic distribution within the multilayer with a conductivity that does not depend on the excitation of electrons from the surface adatoms.

**Figure 3:**
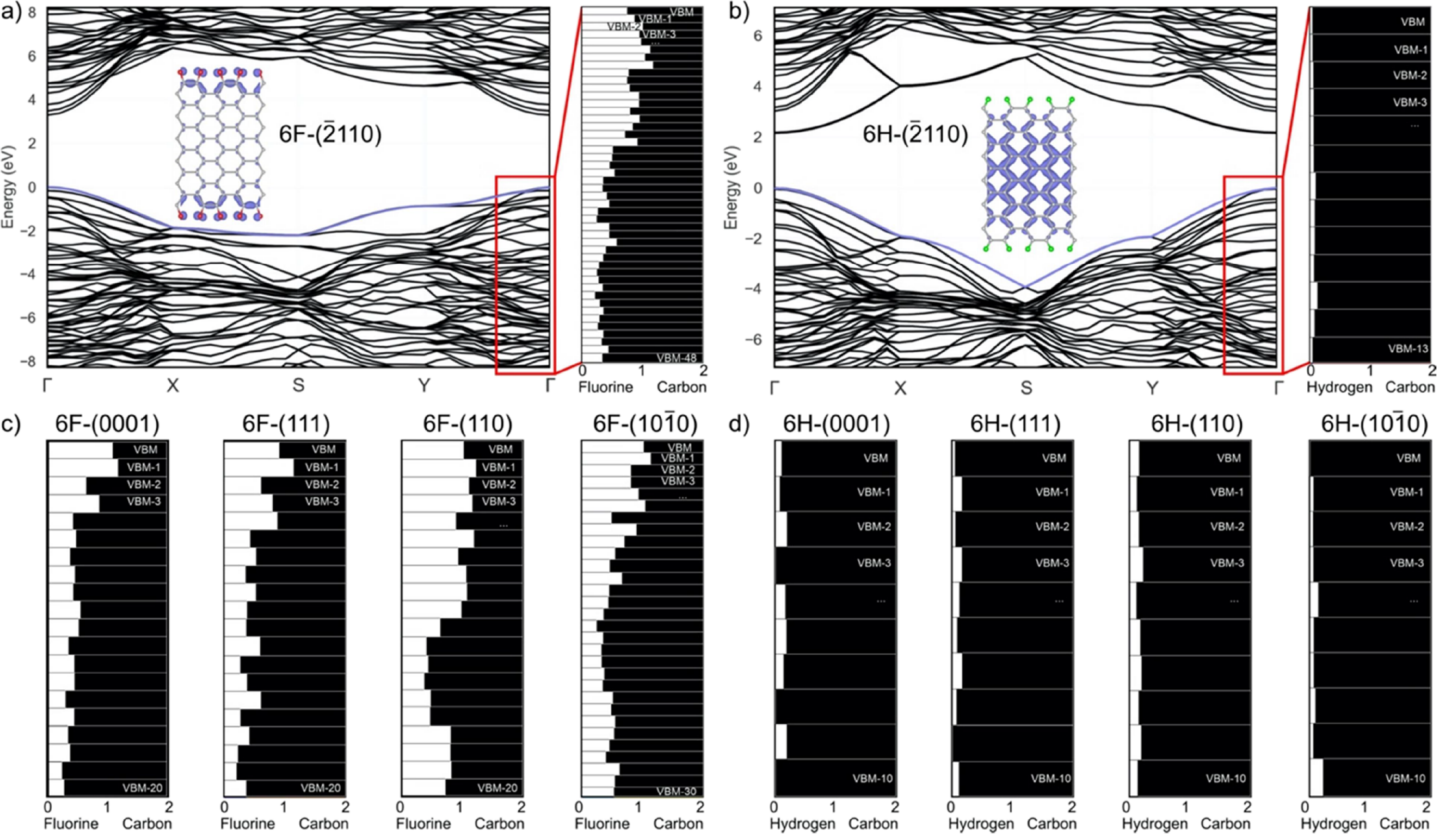
Electronic band structures of six-layer (2̅110) diamanes functionalized by (a) fluorine and (b) hydrogen, calculated with TB09. The insets show the charge density isosurface of electrons localized at the VBM. The isosurface value for the fluorinated diamane is 0.0068 e/Å^3^; for the hydrogenated diamane, the isosurface value is 0.0045 e/Å^3^. The electronic band colored in blue corresponds to the VBM for which the charge density is plotted for both fluorinated and hydrogenated diamanes. The right panels show the ratio of atomic population of fluorine/hydrogen and carbon atoms from the VBM to lower bands. Similar plots are shown for the atomic population for the other crystallographic orientations (111), (110), (0001), and (101̅0) of (c) fluorinated and (d) hydrogenated diamanes from the VBM to the lower bands.

Hence, Bader theory^46^ provides a detailed characterization of the surface and bulk electronic states in diamanes with different functionalizations and provides a partition of charge in different atomic contributions. In such theory (also called QTAIM, i.e., quantum theory of atoms in molecules),^46^ the atoms are not considered as spherical units intrinsically defined and independent by the context, but their shape and volume are strongly influenced by the surrounding atoms. Atoms in molecules are defined as atomic basins delimited by the so-called zero-flux surface. Such surface is made by the infinity of points ***r***, for which the dot product of the gradient of electron density, ∇ρ, and the normal vector to the surface, ***n*^**, is zero (zero-flux boundary condition, ∇ρ(***r***) · ***n*^**(***r***) = 0). We have calculated the charge density at the VBM and integrated such density within Bader atomic basins bounded by zero-flux surfaces calculated with the full electron density.^46^ This was done for the six-layer diamanes. It is seen that hydrogenated diamane states, close to the top of the valence band, are predominantly bulk electronic states, while fluorinated diamanes possess important localized surface electronic states, see Figures 3, S12, and Table S15 in the Supporting Information.

Let us have now a look in detail at the diamane with the (2̅110) surface (Figure 3), which is the characteristic multilayer showing two different band gap behaviors as a function of the functionalization type (fluorine or hydrogen). As can be seen by the KS band structures in Figure 3a,b, where the electronic band structures calculated by TB09 are shown: bulk and surface electronic states are observed for hydrogenated and fluorinated diamanes respectively.

Now, we explain in detail the fluorinated (Figure 3a) and hydrogenated diamanes with (2̅110) surfaces (Figure 3b) to rationalize the correlation between the KS electronic band structure and the band population from the VBM to the inner bands.

The KS electronic band structures of fluorinated diamanes (Figures S2, S4, S6, S8, and S10 in the Supporting Information) show surface electronic states that are supported by the population of VBM and first-inner bands are characterized by surface fluorine atoms and their bonded carbon atoms (Figure 3a). As we look in deeper bands, the contribution of carbon atoms to the electronic population becomes predominant (Figure 3a). The same kind of band population is shown by the Bader analysis for the other structures (Figure 3c).

The KS electronic band structures of hydrogenated diamanes (Figures S1, S3, S5, S7, and S9 in the Supporting Information) show bulk electronic states that are supported by spreading of hydrogen atomic charge density into the inner bands with a charge density at the VBM dominated by the atomic charge density of carbon atoms as shown in Figure 3b,d.

The (2̅110) hydrogenated diamane shows a pronounced difference between conduction band minima (CBM) and the first closest higher conduction band. This is responsible for such different band gap behaviors as seen in Figure 2 with respect to the hydrogenated diamanes with other surfaces showing a similar energy difference between CBM and the higher conduction bands.

**Figure 4:**
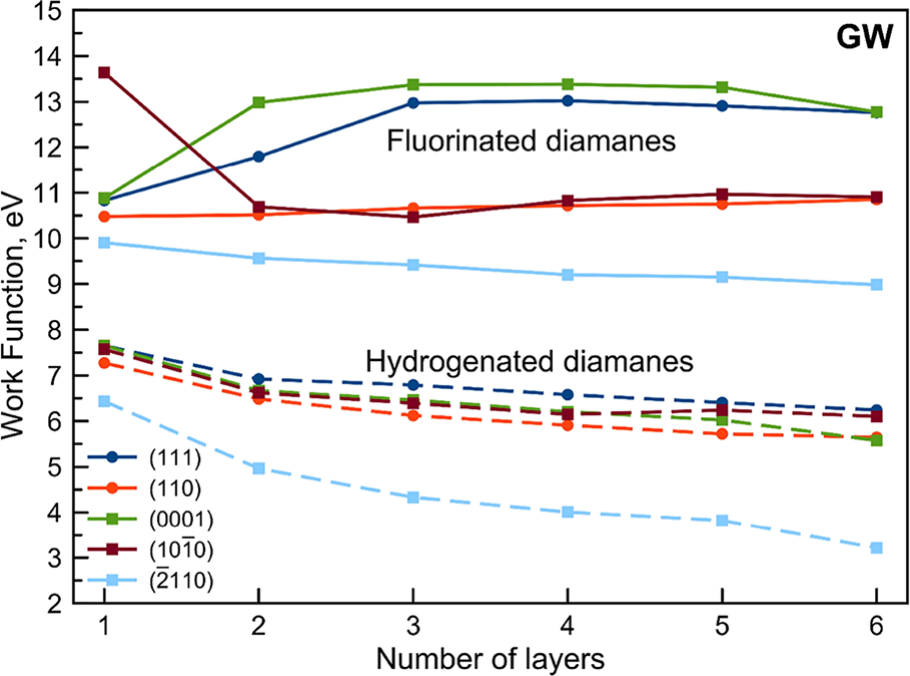
Work function depending on the number of layers for the considered fluorinated and hydrogenated diamanes with different orientations of surfaces obtained by *GW*@PBE.

As the difference between surface and bulk states at band edges affects the properties at the surface in different ways, we have calculated the work function from PBE (see Supporting Information Figure S13 and Table S13) and *GW*@PBE (see Figure 4 and Supporting Information Table S13), depending on the number of layers, to understand how many layers are necessary to obtain a promising FED.

Indeed, we have observed a different work function behavior after *GW* correction that significantly shifts the VBM with respect to PBE results as expected for the band gap results obtained with *GW* correction on top of TB09.

The work function values of fluorinated diamanes are larger than that of hydrogenated ones and linearly decreases except for (111) and (0001) surfaces where the work function displays a behavior similar to the band gap dependence, namely, the work function increases from the monolayer to three layers and then decreases. This is coherent with the formation of local surface states in the case of multilayers, which for such structure also affects the work function behavior. Furthermore, the F-(2̅110) diamanes have the same behavior as H-(111), H-(110), H-(0001), and H-(101̅0) films, although having a higher work function than hydrogenated ones for thin films, see Figure 4. It monotonically decreases with the increase of the number of layers, reaching ∼6 eV at six layers.

The *GW* correction singles out the hydrogenated (2̅110) surface that has the lowest work function values, starting from ∼6.5 eV for the monolayer, decreasing with the number of layers, and achieving ∼3.5 eV for six layers.

An additional concept that we analyzed in our work is the dipole moment in 2D materials to understand the intrinsic electronic properties of diamanes. Usually, in 2D materials one evaluates the surface dipole moment, which describes the transfer of charge that happens at the interface between the edge of the material and vacuum when a fictitious potential is applied. To describe the charge distribution at the surface, different models were previously developed: the Helmholtz model, where an electric double layer consisting of two oppositely charged layers is assumed and where the charges on the surface of the material form a pearl necklace (i.e., uniform distribution) where charges are free to move;^54^ the Gouy–Chapman model, where the interfacial potential is created like in the Helmholtz model, with the difference that the charges in vacuum are not free to move and are in the same number and opposite in sign to those of the surface of the material;^55,56^ and the Stern model, which suggests a hybrid model between the two previously described, with ions that have finite size, so they cannot approach the surface closer than a few nanometers.^57^

Unfortunately, these classical models fail to describe the experimental data and cannot describe the formation of dipole moments at the interface. Furthermore, these kinds of models do not deliver any intrinsic information about the electronic properties of materials.

Now, if we focus on the effects on the electronic properties simply due to the functionalization of the surfaces of diamanes, then the chemical bonding between carbon and surface adatoms will be characterized by the formation of a bond dipole moment, whose intensity depends on the different electronegativities of elements. These bond dipole moments appearing between carbon and surface adatoms will be responsible for the charge separation at the surface of the film determining the intrinsic electronic properties of that functionalized material. Thus, the direction of each bond dipole moment at the surface will characterize the chemical properties of the material at the atomistic level. If a bond dipole moment between carbon and surface adatoms is directed toward the deep layers or out from the material, then the electron affinity of the surface will change together with the chemical reactivity.

Hence, as we know, the bond dipole moment is defined as^58^
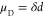
1where δ is the difference between the partial charges of bounded atoms, δ = δ^+^ – δ^–^, *d* is the bond length in angstroms.

The problem with this historical definition stems from the estimation of atomic partial charges. At present, such estimation relies on using first-principles calculations that are nontrivial due to the self-interaction error and requires complex calculations within constrained DFT^59−61^ and the kind of chosen atomic partition can also be a source of errors as underestimation or overestimation.^60^ It would be desirable to determine the bond dipole moments a priori from a simpler perspective, in order to predict more straightforwardly new functionalized multilayers with desired characteristics that strictly depend on the electron density distribution at the surface.

Thus, we have tested the possibility to correlate the difference between the electronegativities of two atoms, Δ*X*_AB_, with their supposed bond dipole moment. Here, we have looked for a correlation between the Δ*X*_AB_ coming from the Tantardini–Oganov^62^ thermochemical electronegativity scale and bond dipole moment. We estimate the bond dipole moment in terms of Δ*X*_AB_ as:
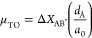
2where Δ*X*_AB_ is multiplied by the bond distance in angstroms, *d*_Å_, and divided by the Bohr radius, *a*_0_, in angstroms, which is equal to 0.529177 Å. This allows us to use dimensionless values for bond dipole moments.

We have tested our formula on molecules containing hydrogen atoms to see if our approach is applicable. Since we selected only neutral diatomic molecules, where δ^+^ is by nature equal to −δ^–^, and the estimated bond dipoles μ_TO_ may be rigorously compared with the experimental or calculated dipole moments. The latter, obtained from the calculated wavefunction as an average of the ***r*** operator, may be also roughly estimated using atomic charges obtained from the same wavefunction and using some basis set or real space partition criterion. Results shown in Table 1 confirm our suggestion to use μ_TO_ as a semiquantitative predictor for (bond) dipole moment.

**Table 1:** Bond Length, Electronegativity Difference, Calculated and Measured Dipole Moment for Several Molecules with Hydrogen^a^

diatomic molecule	*d*_*Å*_	Δ*X*_AB_	μ_TO_	μ_D_/(*D*)	μ_wf_/(*D*)	μ_Bader_(CC)/(*D*)	μ_Bader_(VASP)/(*D*)^62^
HF	0.9200	0.96	1.67	1.82^63^	1.93	3.56	3.18^62^
HCl	1.2700	0.5	1.20	1.08^63^	1.19	1.95	2.32^62^
HBr	1.4100	0.41	1.09	0.82^63^	0.89	1.15	2.84^62^
HI	1.6100	0.16	0.49	0.44^63^	0.52	0.85	3.40^62^

a μ_Bader_(CC) is calculated in this work, while μ_Bader_ (VASP) is from ref (62).

The value of μ_TO_ is seen within 10–30% of those taken from the NIST database^63^ corresponding to experimental measured values in debye, μ_D_, and within 10–20% of those obtained from our coupled cluster wavefunction calculations (μ_wf_) using single, double, and triple excitations and a triple ζ local basis set. Dipole moments simply approximated from Bader charges with a plane wave basis set in the VASP package (μ_Bader_(VASP) from ref (62)) greatly overestimate the experimental ones. Though a bit closer to the NIST database values, the dipole moments calculated from Bader charges obtained from the coupled cluster wavefunction are much larger than both those from the experiment and those calculated directly from the wavefunction. This is not at all surprising since to reconstruct exactly the wavefunction dipole moment, one has to add to the considered charge transfer contribution also that due to the atomic dipoles that in the case of systems with large ionic character is opposite in sign and of the same order of magnitude as the charge transfer contribution.^64,65^

It is noteworthy that it is possible to introduce a “semiquantitative” instrument to determine a priori the bond dipole moment by simply using a pen and paper with Tantardini–Oganov electronegativity without complex quantum chemical calculations.

Thus, by simply knowing the geometry of the system and the difference of electronegativity between two bound atoms, it is possible to define their bond dipole moment. In fact, the distance between two bound atoms assumes the meaning of weight of electronic cloud distribution between two atoms.

We have then estimated μ_TO_ between carbon and hydrogen/fluorine atoms for our diamanes, showing that in case of surface and bulk states for hydrogenated and fluorinated diamanes, respectively, the dipole moment does not change with increasing the number of layers (see Tables S11 and S12 in the Supporting Information). However, if we define the surface dipole moment as the average of μ_TO_ between the adatoms of the surface and the surface carbon atoms, then we can describe the polarization at the surface after functionalization.

Here, we observed that the average of μ_TO_ is ∼2.5 for all fluorinated diamanes, while it is only ∼0.2 for hydrogenated ones. The strongest dipole moment present on the fluorinated diamane surfaces can be also considered responsible for the linear behavior of the work function and in the observed range, the saturation is not achieved. Nevertheless, the lowest surface dipole moment of hydrogenated diamanes allows them to achieve faster saturation, making them a better candidate for FEDs.

## Conclusions

Our first-principles investigation of the direct and fundamental band gaps of hydrogenated and fluorinated diamanes, which represent two successful cases of reversible chemical doping, showed a behavior compatible with the formation of occupied surface electronic states for hydrogenated diamanes but no such surface states for fluorinated diamanes were observed. The Bader analysis allowed us to corroborate this view through the study of the electronic population at the VBM. The nature of band gap behavior is responsible for the changes of the work function, namely, bulk electronic states in hydrogenated diamanes are correlated with the small values of the work function that rapidly saturates with the increase of the number of layers, while surface electronic states in fluorinated diamanes deliver a large work function that is also observed to decrease slowly with increasing the number of layers with the exception of fluorinated (110) and (2̅110). It is noteworthy that H-(2̅110) is the most suitable surface for FEDs. We have also been able to correlate the Tantardini–Oganov electronegativity scale with the bond dipole moment showing the extension of such an approach to condensed matters and envisaging the possibility to avoid expensive first-principles calculations and a priori making a prediction on surface reactivity.

## Methods

### Computational Details

Structure relaxations and total energy calculations were performed using the optimized norm-conserving Vanderbilt pseudopotentials (ONCVP)^66,67^ and the GGA with the Perdew–Burke–Ernzerhof (PBE) exchange-correlation density functional^68^ as implemented in ABINIT ver. 9.0.4.^69,70^ ONCVP were adopted with 4, 1, and 7 valence electrons for carbon, hydrogen, and fluorine atoms, respectively. The geometry optimization relies on the Broyden–Fletcher–Goldfarb–Shanno (BFGS)^71−74^ algorithm with a convergence cutoff of 5.0 × 10^–5^ Ha/bohr for the maximum net force on atoms, while the self-consistent field convergence criteria is based on a residual potential cutoff equal to 10^–12^ Ha. A plane wave energy cutoff of 50 Ha and Fermi–Dirac smearing of electronic occupations equal to 0.001 Ha ensure the convergence of total energies. The Γ-centered *k*-point meshes of 6 × 6 × 1 for diamanes and 6 × 6 × 6 for bulk diamond and lonsdaleite were used for the first Brillouin zone sampling.

*G*_0_*W*_0_ and KS electronic band structure calculations were performed with norm-conserving relativistic separable dual-space Gaussian pseudopotentials (HGH),^75^ having the same number of valence electrons adopted in the ONCVP. KS electronic structures were calculated using TB09 and PBE exchange-correlation functionals.^50^ For *G*_0_*W*_0_ calculations, the number of unoccupied bands per atom is 50 and the energy cutoff for the dielectric matrix is 5 Ha, which guarantees a band gap convergence in the order of 2 meV. *G*_0_*W*_0_ calculations for the multilayers were performed considering the Coulomb singularity problem that happens at ***G*** = 0 and that hinders the convergence with respect to the number of *k*-points used to sample the first Brillouin zone, thanks to the Ismail-Beigi methodology.^76^ These calculations were performed considering the degeneracy of bands at the VBM and CBM looking for the *GW*@TB09 band gap as the difference between the two degenerate bands at VBM and the two degenerate bands at the CBM.

No attempt was made to perform self-consistent *GW* calculations. Instead, calculations relied on the DFT electronic charge densities throughout. The electronic charge density change from such self-consistent *GW* with respect to the DFT one is not large anyhow, the variation being in the order of a millielectron per atomic unit, as reported in ref (77). Thus, electronic charge densities at the VBM showed throughout are KS TB09 ones.

The planewave-based multilayer calculations, with an inherent artificial periodicity perpendicular to the multilayer, were performed without adding a planar dipole layer in the vacuum region,^78^ because all the slabs are hydrogenated (or fluorinated) on both sides of the slab, equally, so, their surface dipole long-range effects cancel each other and there is no long-range buildup of the electric field due to these surface dipoles in the ground-state.

The work function was determined as the difference of energy between the highest occupied state and the vacuum level, the latter obtained by the macroscopic average technique of Baldereschi et al.^79^ The highest state energy was the one from *GW*@PBE calculation, following the so-called *GW*-VBM approach, see eq 3 of ref (80). Results were compared with the uncorrected PBE values in the Supporting Information.

The electronic charge density for test molecules (see Table 1) was calculated with coupled cluster single, double, and triple excitations using Gaussian local basis sets aug-cc-pVTZ (all-electron triple ζ basis set with diffusive and polarized) for hydrogen, fluorine, and chlorine atoms,^81^ while aug-cc-pwCVTZ-PP (relativistic triple ζ basis set with diffusive and polarized) was used for bromine and iodine atoms.^82,83^ Their molecular geometries were taken from experimental data^63^ and are shown in the Table 1 of the main text together with experimental dipole moments. The threshold energy of SC convergence was chosen to be 10^–8^ Ha.

All first-principles and quasiparticle calculations on solids were performed with ABINIT.^69,70^ The post-Hartree–Fock calculations on molecules were performed using Gaussian G16 version^84^ The Bader analysis was performed using the CRITIC2 program.^85^ The crystal structures were visualized using VESTA software.^86^

## Supporting Information Available

It contains details of the atomic structure of the studied diamanes films, the electronic band structures, the dependency of direct and fundamental band gaps as a function of the layer thickness, calculated by using the TB09 approach, and the charge density coming from various atomic basins at the valence band maximum and inner bands (PDF)
