# Supplementary material for: Electronic Properties of Functionalized Diamanes for Field-Emission Displays
Source: ACS Appl Mater Interfaces. 2023 Mar 16;15(12):16317–26. doi: 10.1021/acsami.3c01536 (PMC10064316; doi:10.1021/acsami.3c01536)
Supplement: Supplementary file 1 — am3c01536_si_001.pdf [file am3c01536_si_001.pdf]

# Supporting Information

# Electronic Properties of Functionalized Diamanes for Field Emission Displays

*Christian Tantardini*[*,1,2,3], *Alexander G. Kvashnin*[*,4], *Maryam Azizi*[5], *Xavier Gonze*[*,5], *Carlo Gatti*[6], *Tariq Altalhi*[7], *Boris I. Yakobson*[*,2,7]

[1]*Hylleraas Center, Department of Chemistry, UiT The Arctic University of Norway, P.O. Box 6050 Langnes, N-9037 Troms., Norway*

[2]*Department of Materials Science, Rice University, Houston, Texas 77005, United States of America.*

[3]*Institute of Solid State Chemistry and Mechanochemistry SB RAS, 630128, Novosibirsk, Russian Federation.*

[4]*Skolkovo Institute of Science and Technology, 121205, Bolshoi Blv. 30, Building 1, Moscow, Russian Federation.*

[5]*Université catholique de Louvain, Place de l'Université 1, 1348, Ottignies-Louvain-la-Neuve, Belgium.*

[6]*CNR - Consiglio Nazionale delle Ricerche, SCITEC - Istituto di Scienze e Tecnologie Chimiche "Giulio Natta", sezione di via Golgi, 19, 20133 Milan, Italy.*

[7]*Chemistry Department, Taif University, Al Hawiyah, Taif 26571, Saudi Arabia.*

Correspond to: Ch.T, christiantantardini@ymail.com; A.G.K., a.kvashnin@skoltech.ru; X.G, xavier.gonze@uclouvain.be; B.I.Y., biy@rice.edu.

The present supporting information contains:

(i) Figures S1-S10. Electronic band structures of hydrogenated and fluorinated diamanes for different thicknesses, for the five crystallographic orientations of surfaces calculated by using TB09

(ii) Figure S11. Dependency of direct and fundamental electronic gaps on the inverse square of number of layers for considered diamanes

(iii) Figure S12. Charge density of the valence band maximum for 6-layer films for considered diamanes

(iv) Figure S13. Work function as a function of number of layers calculated by DFT-PBE

(v) Tables S1-S10. Atomic coordinates and Bader's charge for 6 layers of considered diamanes

(vi) Tables S11-S12. Structural and electronic properties of considered diamanes

(vii) Table S13. Calculated highest occupied state energy, potential and work function for considered diamanes with DFT-PBE and GW@PBE

(viii) Table S14. Thickness of considered diamanes

(ix) Table S15. Electronic populations at VBM and levels below projected on carbon and hydrogen/fluorine of considered diamanes

# ELECTRONIC BAND STRUCTURES OF DIAMANES

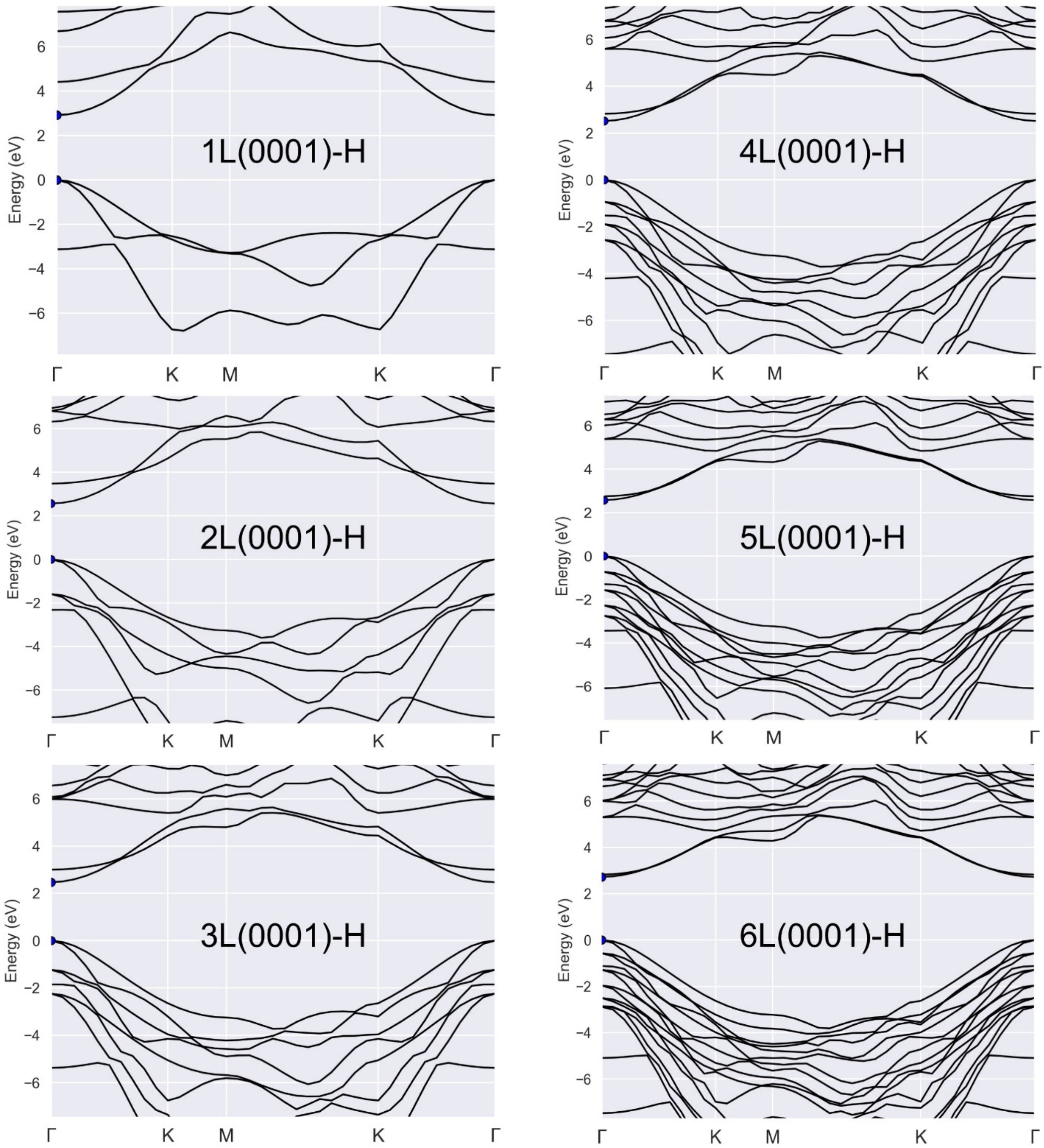


**Figure S1.** Band structures of hydrogenated films with (0001) surface with different thickness from 1 to 6 layers (number of layers) calculated by using TB09.

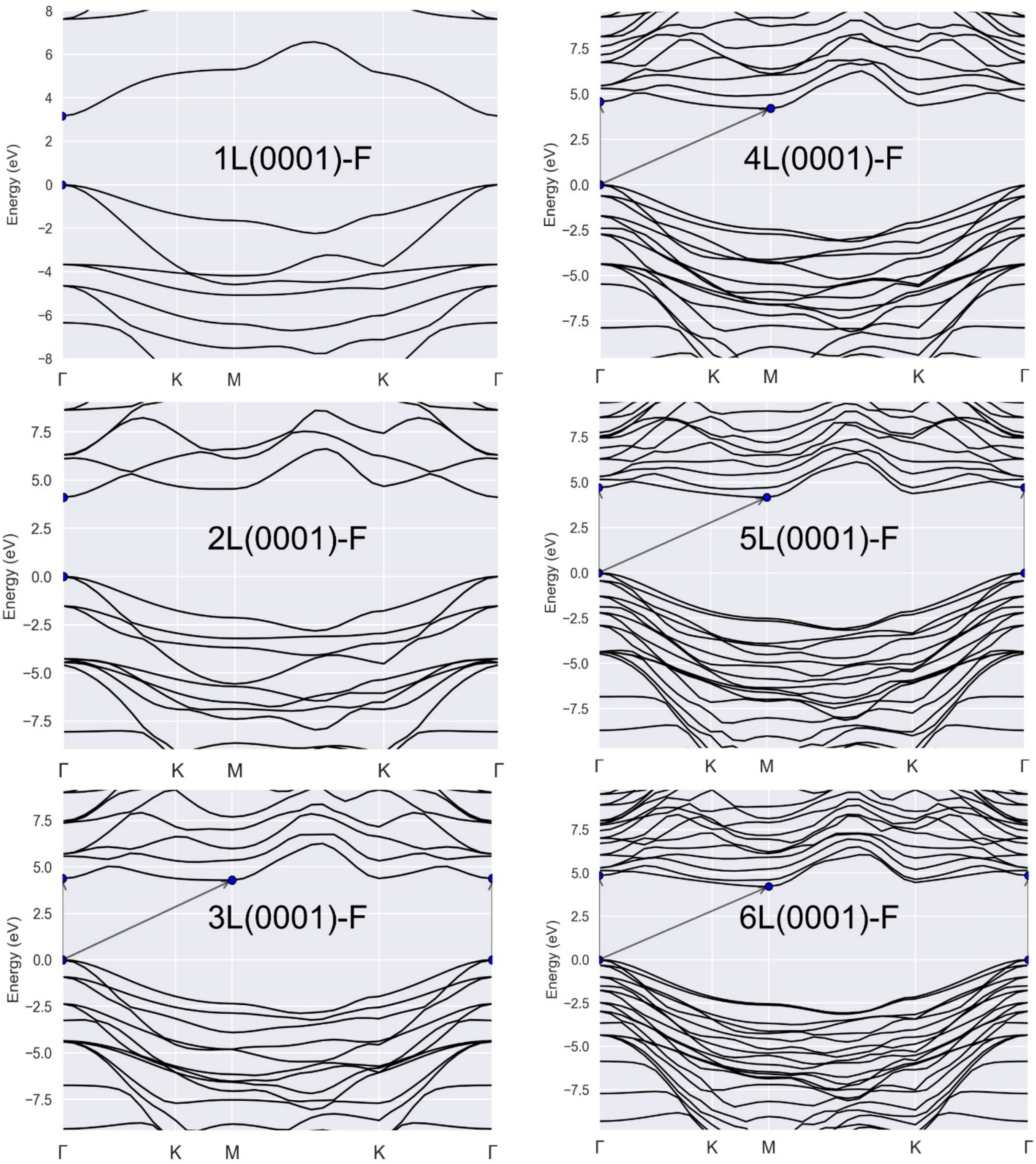


**Figure S2.** Band structures of fluorinated films with (0001) surface with different thickness from 1 to 6 layers (number of layers) calculated by using TB09.

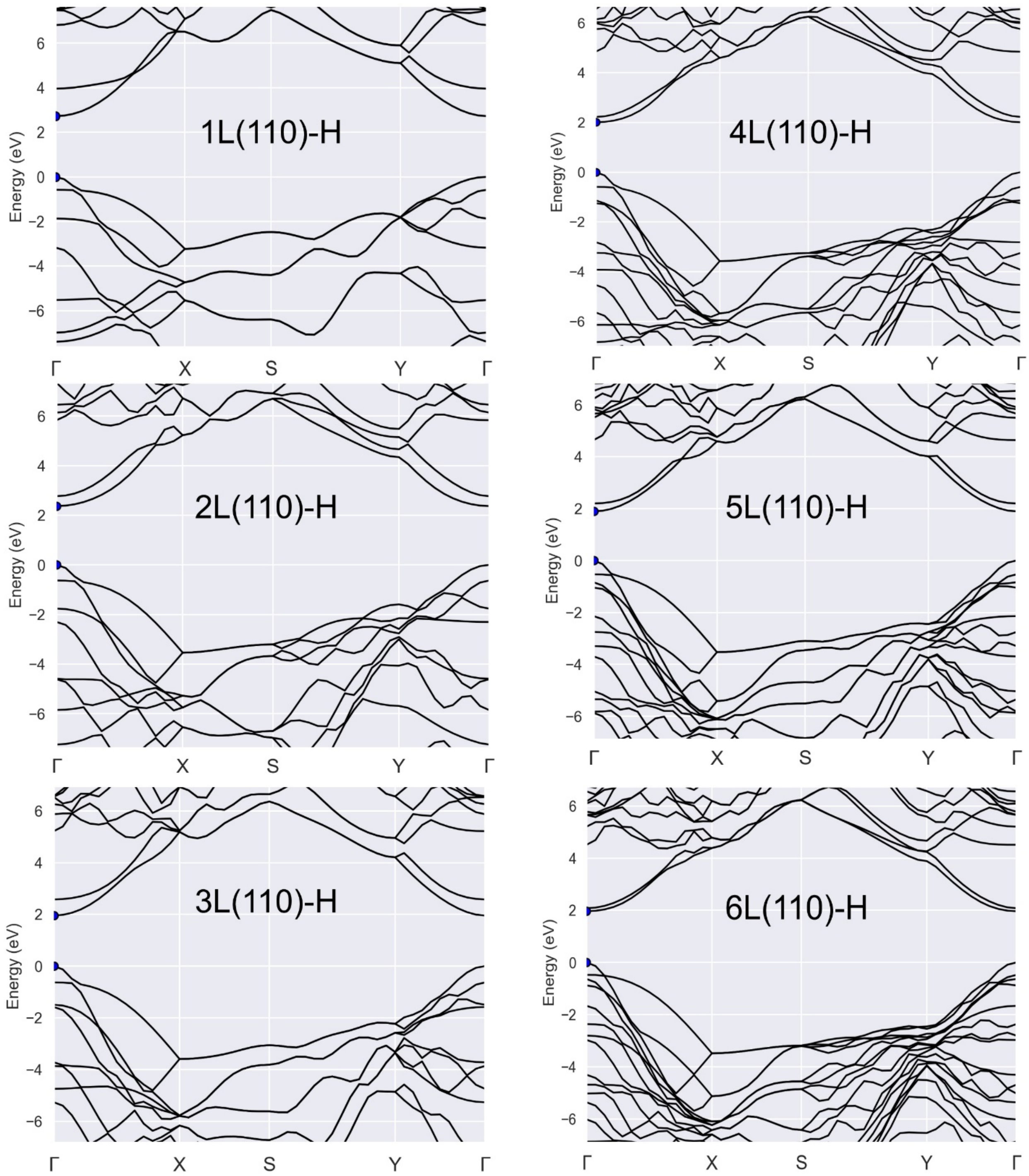


**Figure S3.** Band structures of hydrogenated films with (110) surface with different thickness (number of layers) calculated by using TB09.

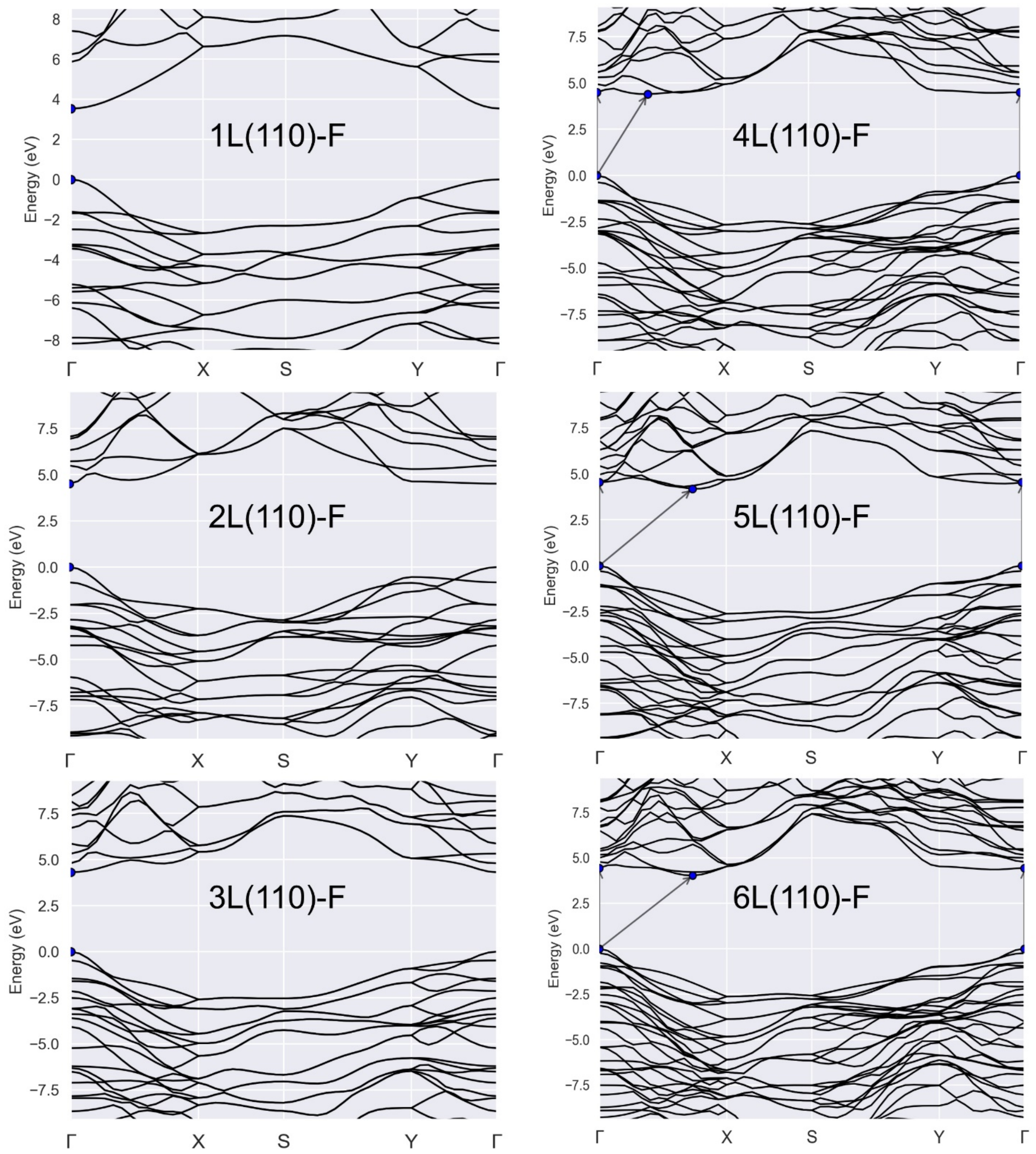


**Figure S4.** Band structures of fluorinated films with (110) surface with different thickness (number of layers) calculated by using TB09.

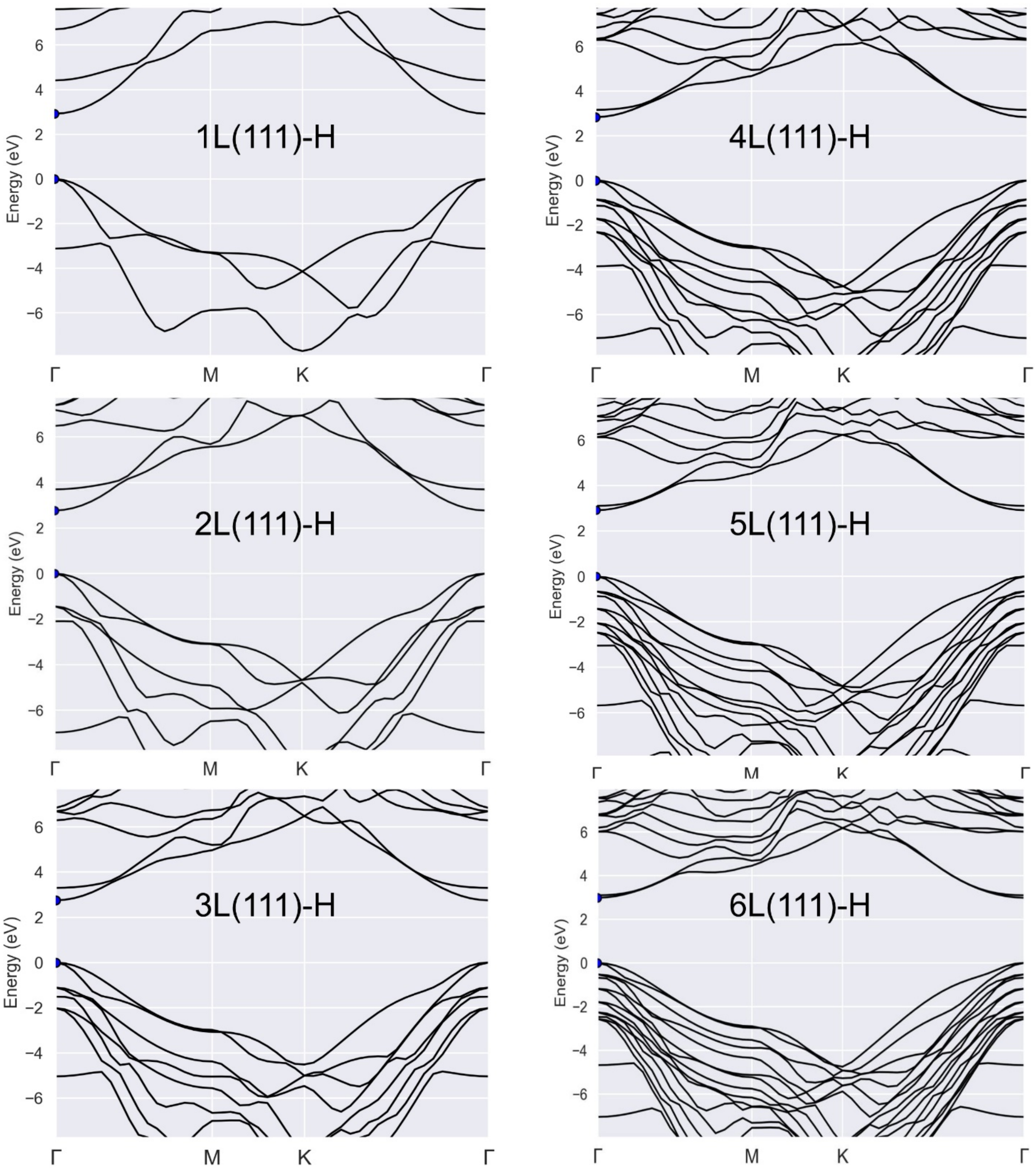


**Figure S5.** Band structures of hydrogenated films with (111) surface with different thickness (number of layers) calculated by using TB09.

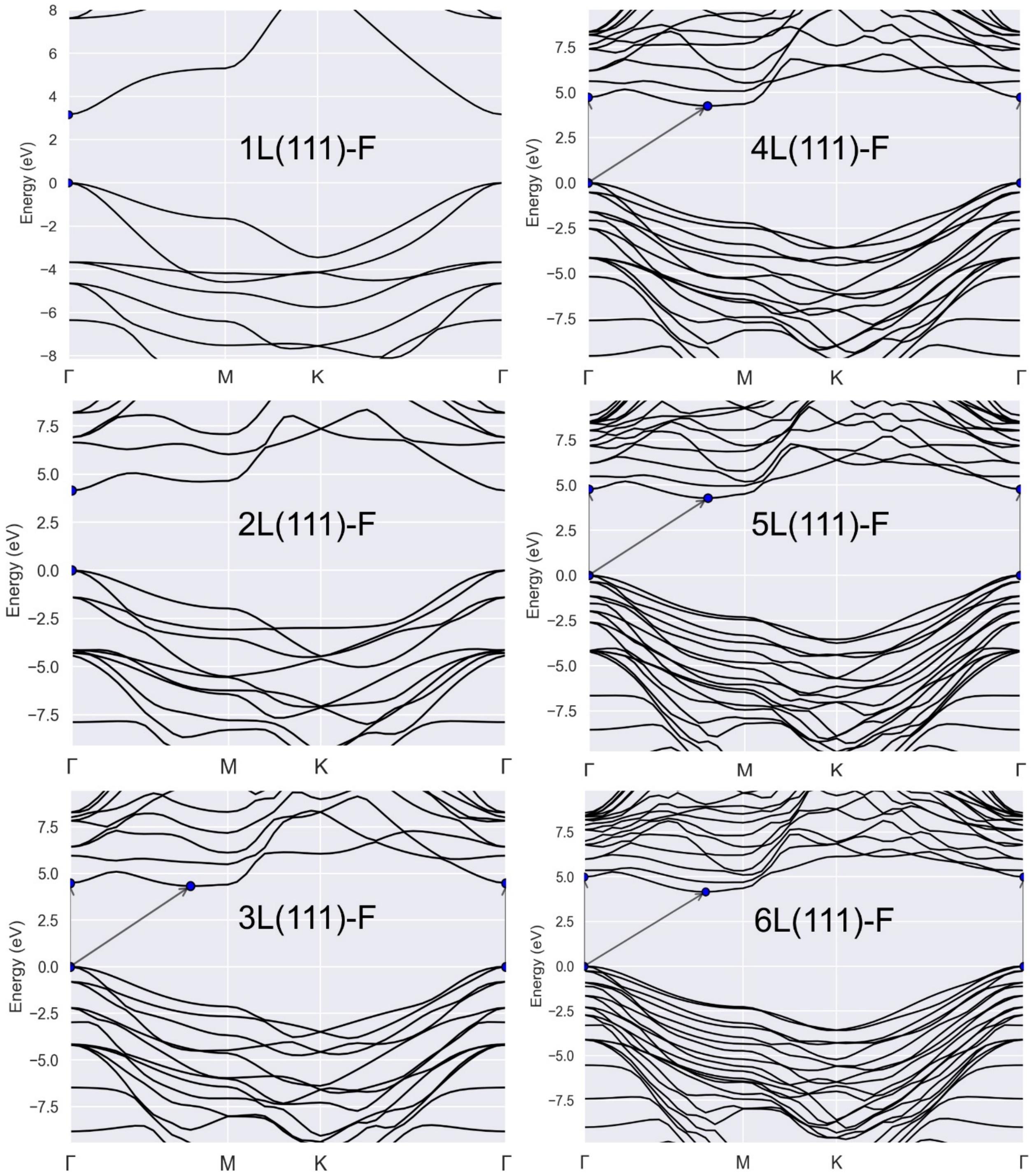


**Figure S6.** Band structures of fluorinated films with (111) surface with different thickness (number of layers) calculated by using TB09.

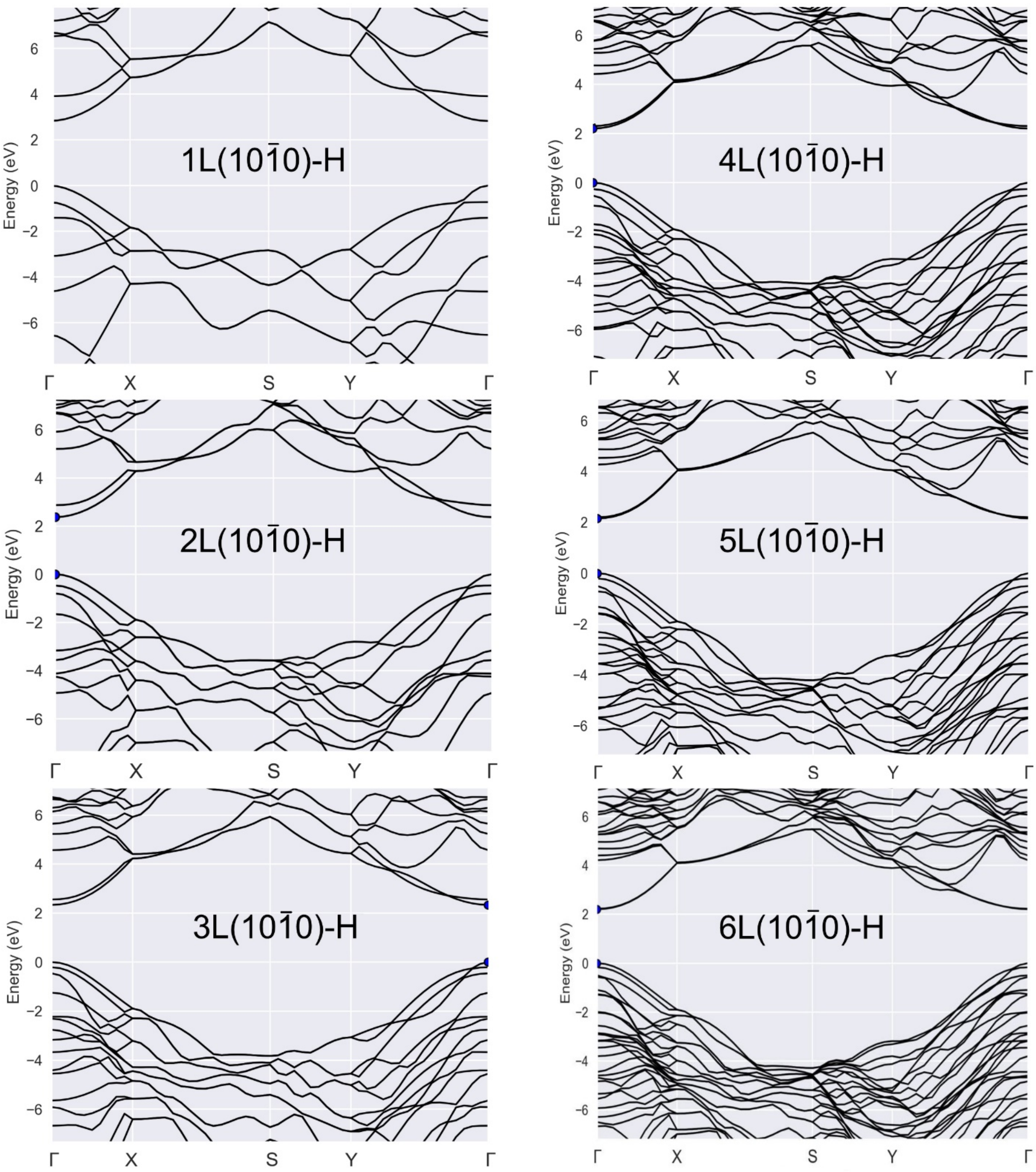


**Figure S7.** Band structures of hydrogenated films with (10$\bar{1}$0) surface with different thickness (number of layers) calculated by using TB09.

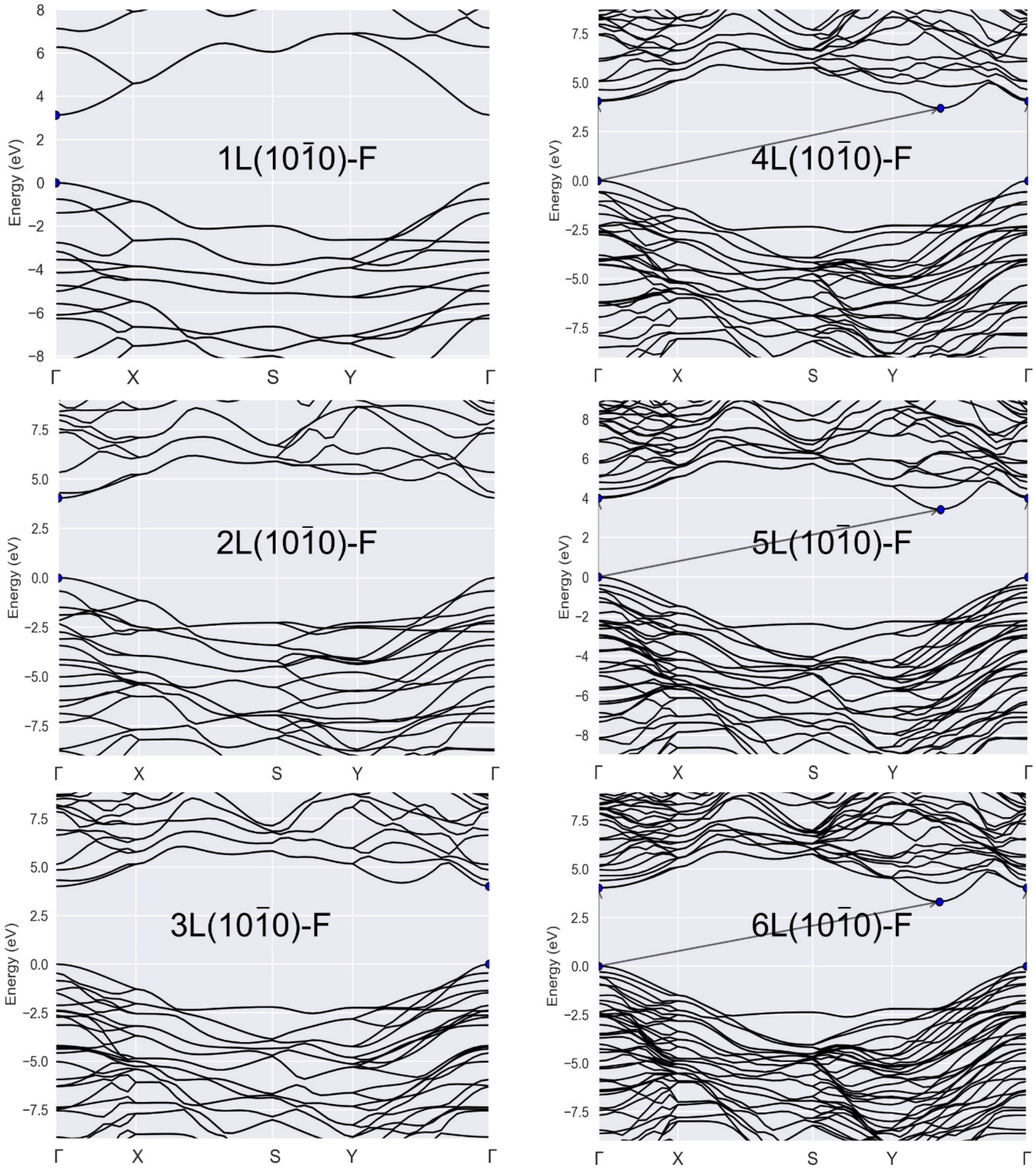


**Figure S8.** Band structures of fluorinated films with $(10\bar{1}0)$ surface with different thickness (number of layers) calculated by using TB09.

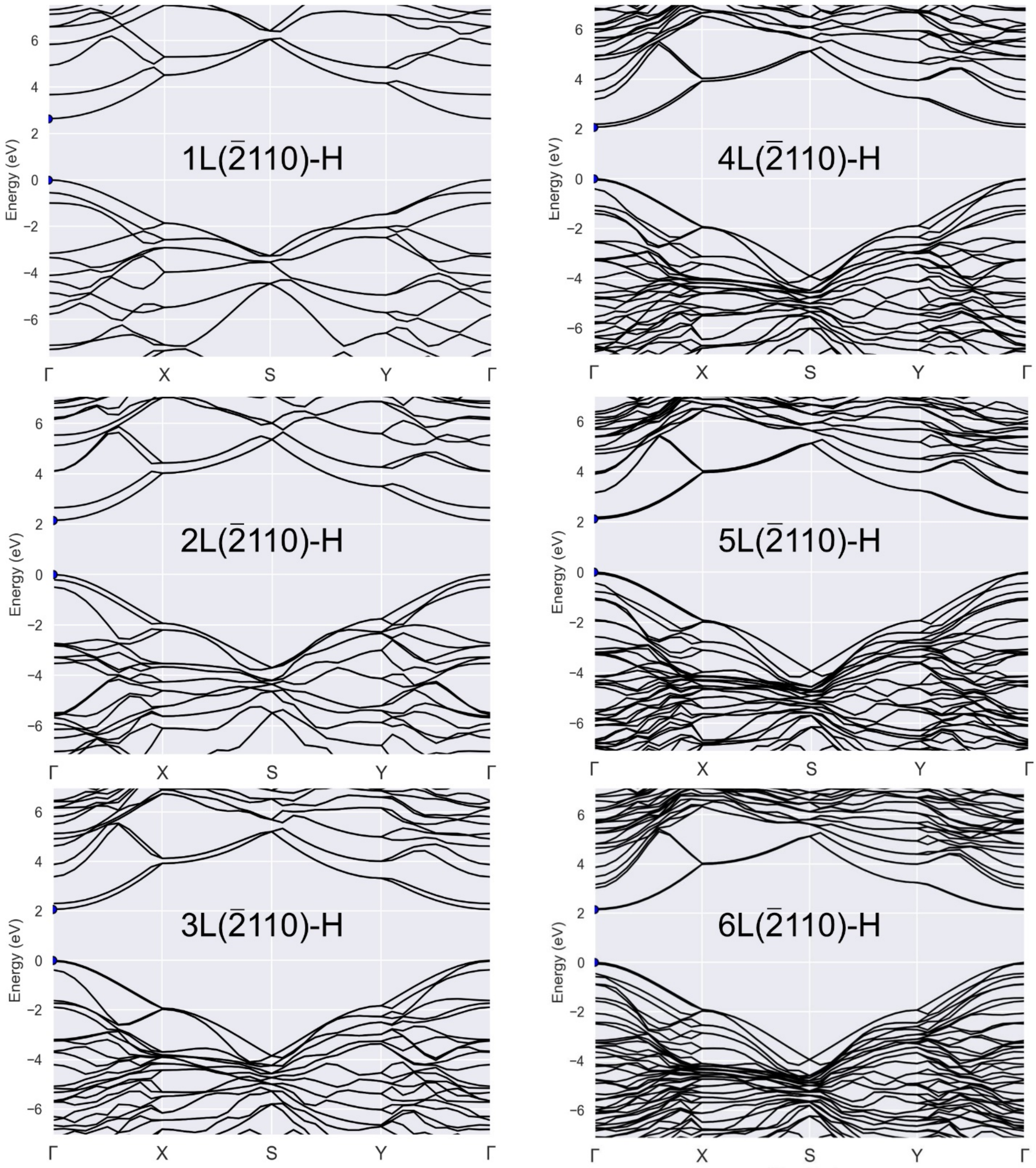


**Figure S9.** Band structures of hydrogenated films with $(2\bar{1}10)$ surface with different thickness (number of layers) calculated by using TB09.

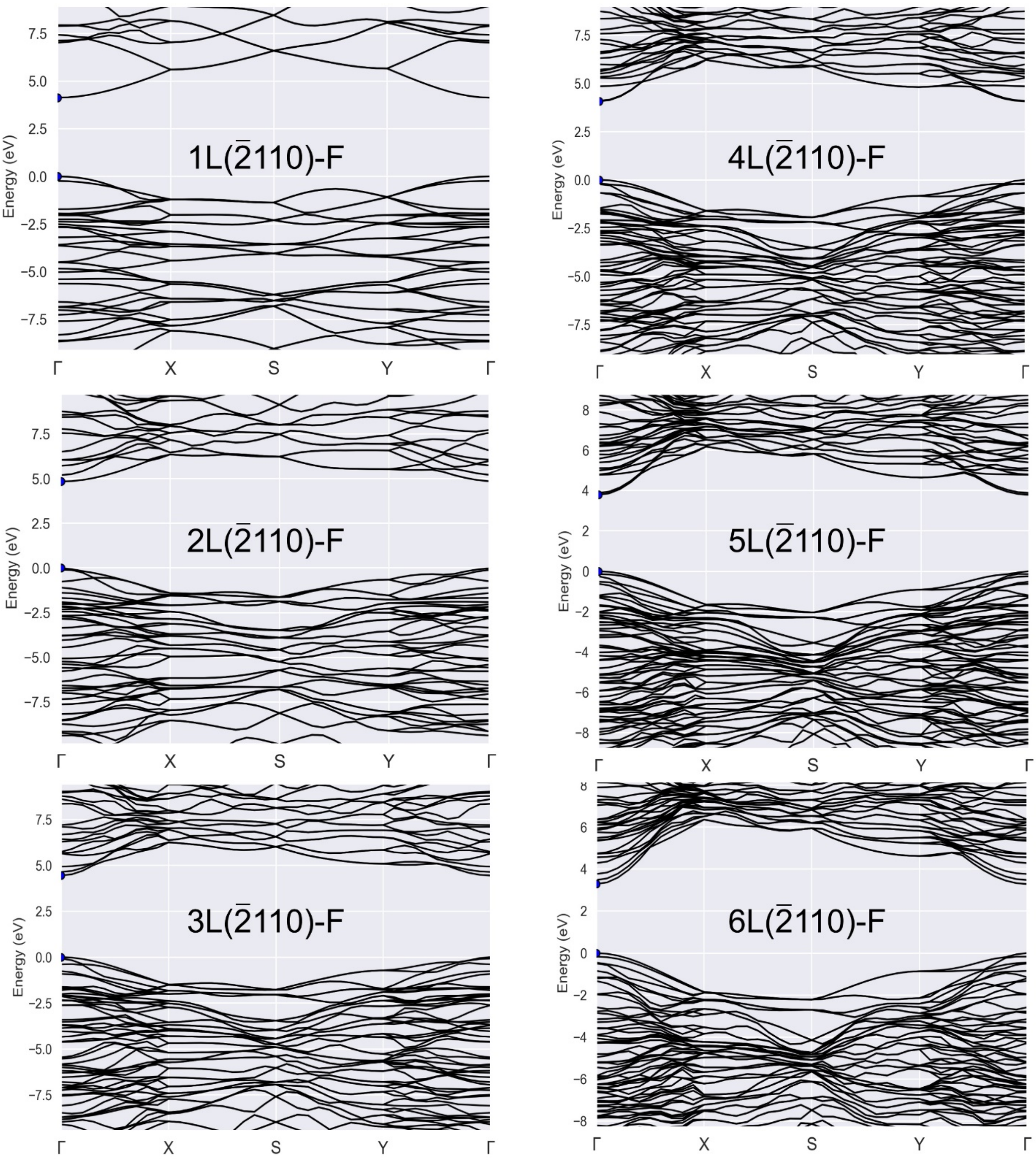


**Figure S10.** Band structures of fluorinated films with ($21\bar{1}0$) surface with different thickness (number of layers) calculated by using TB09.

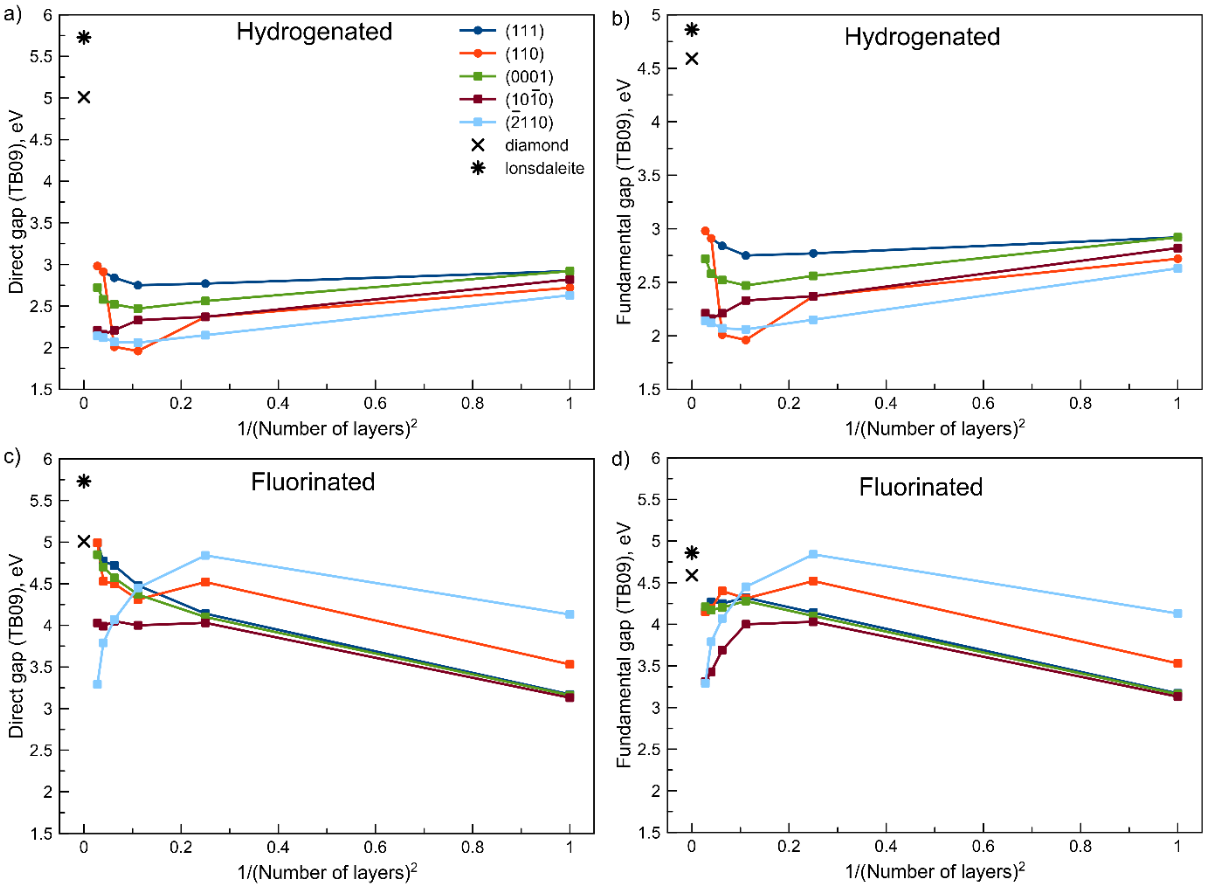


**Figure S11.** Dependency on the squared inverse number of layers (thickness) of the direct - a), c) - and fundamental - b), d) - band gap for hydrogenated - a), b) - and fluorinated - c), d) - diamanes, calculated by using TB09 approach.

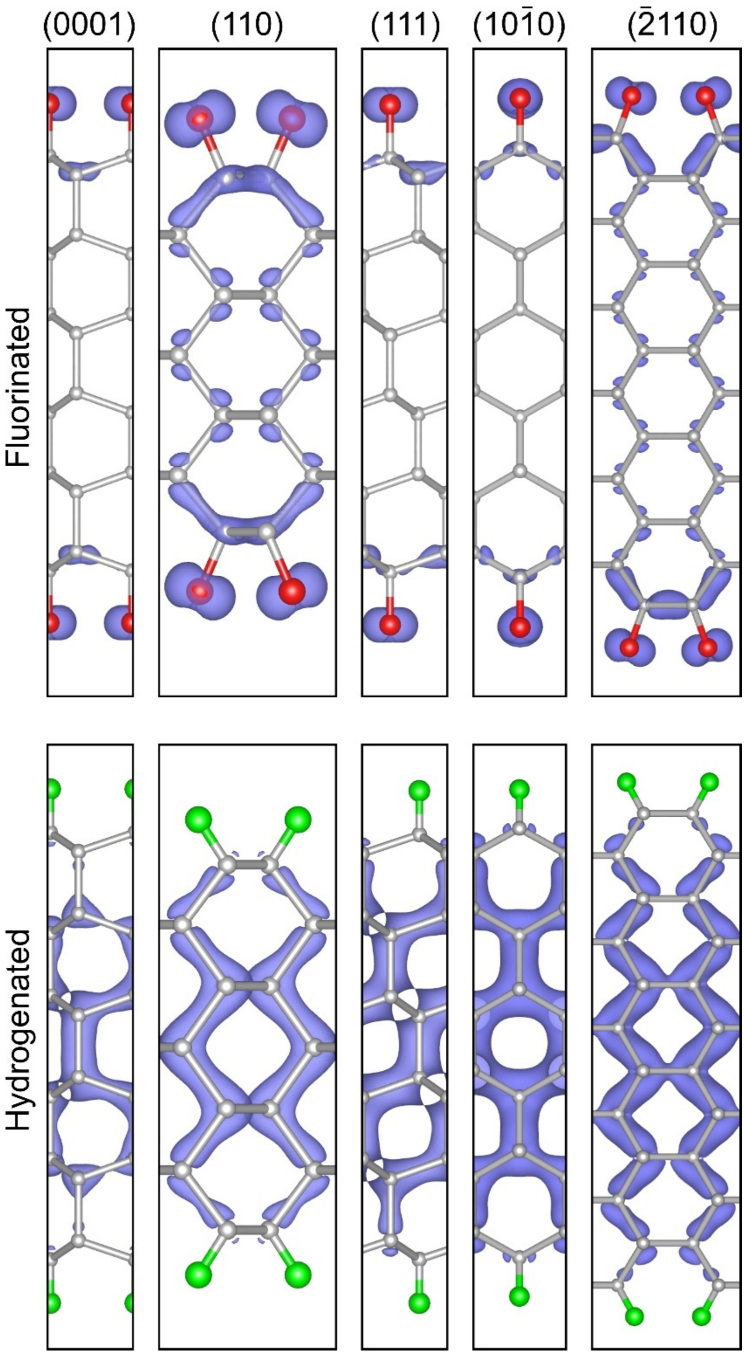


**Figure S12.** Charge density at the valence band maxima for hydrogenated and fluorinated six-layers diamanes.

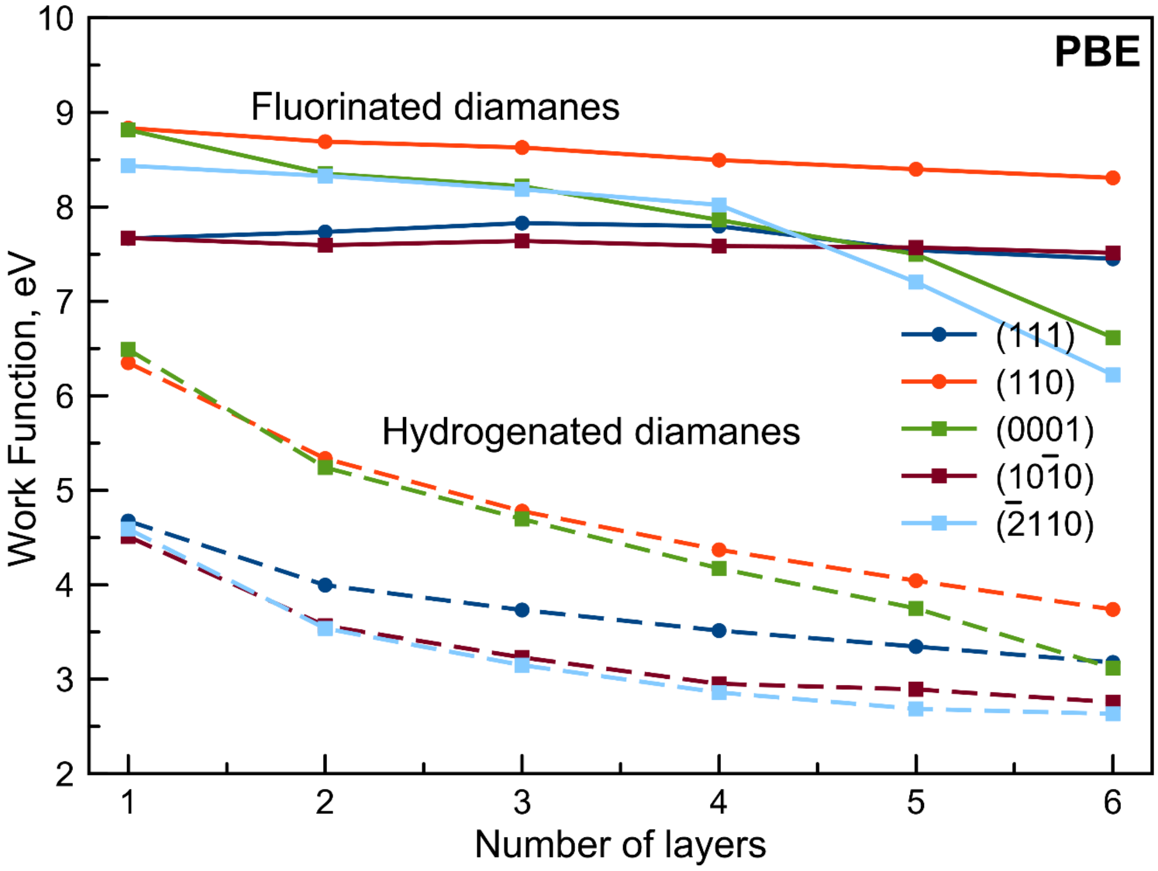


**Figure S13.** Work function depending on the number of layers for considered fluorinated and hydrogenated diamanes with different orientation of surfaces obtained by PBE

**Table S1.** Information about F-(0001) films: atomic coordinates and lattice vectors.

| 1 layer | | | |
|---|---|---|---|
| Atom | X | Y | Z |
| C | 0.0000 | 0.0000 | 0.4935 |
| C | 0.3333 | 0.3333 | 0.5065 |
| F | 0.3333 | 0.3333 | 0.5430 |
| F | 0.0000 | 0.0000 | 0.4570 |
| Tx | 4.9266 | 0.0000 | 0.0000 |
| Ty | 2.4633 | 4.2666 | 0.0000 |
| Tz | 0.0000 | 0.0000 | 71.0681 |
| 2 layers | | | |
| Atom | X | Y | Z |
| C | 0.0000 | 0.0000 | 0.4176 |
| C | 0.3333 | 0.3333 | 0.4304 |
| C | 0.3333 | 0.3333 | 0.4696 |
| C | 0.0000 | 0.0000 | 0.4824 |
| F | 0.0000 | 0.0000 | 0.5165 |
| F | 0.0000 | 0.0000 | 0.3835 |
| Tx | 4.8388 | 0.0000 | 0.0000 |
| Ty | 2.4194 | 4.1905 | 0.0000 |
| Tz | 0.0000 | 0.0000 | 75.5890 |
| 3 layers | | | |
| Atom | X | Y | Z |
| C | 0.0000 | 0.0000 | 0.4052 |
| C | 0.3333 | 0.3333 | 0.4167 |
| C | 0.3333 | 0.3333 | 0.4524 |
| C | 0.0000 | 0.0000 | 0.4643 |
| C | 0.0000 | 0.0000 | 0.4999 |
| C | 0.3333 | 0.3333 | 0.5115 |
| F | 0.3333 | 0.3333 | 0.5426 |
| F | 0.0000 | 0.0000 | 0.3741 |
| Tx | 4.8094 | 0.0000 | 0.0000 |
| Ty | 2.4047 | 4.1651 | 0.0000 |
| Tz | 0.0000 | 0.0000 | 83.1479 |
| 4 layers | | | |
| Atom | X | Y | Z |
| C | 0.0000 | 0.0000 | 0.4264 |
| C | 0.3333 | 0.3333 | 0.4375 |
| C | 0.3333 | 0.3333 | 0.4716 |
| C | 0.0000 | 0.0000 | 0.4830 |
| C | 0.0000 | 0.0000 | 0.5170 |
| C | 0.3333 | 0.3333 | 0.5284 |
| C | 0.3333 | 0.3333 | 0.5625 |
| C | 0.0000 | 0.0000 | 0.5736 |
| F | 0.0000 | 0.0000 | 0.6033 |
| F | 0.0000 | 0.0000 | 0.3967 |
| Tx | 4.7946 | 0.0000 | 0.0000 |
| Ty | 2.3973 | 4.1523 | 0.0000 |
| Tz | 0.0000 | 0.0000 | 86.9274 |

| 5 layers | | | |
|---|---|---|---|
| Atom | X | Y | Z |
| C | 0.0000 | 0.0000 | 0.4077 |
| C | 0.3333 | 0.3333 | 0.4184 |
| C | 0.3333 | 0.3333 | 0.4510 |
| C | 0.0000 | 0.0000 | 0.4619 |
| C | 0.0000 | 0.0000 | 0.4945 |
| C | 0.3333 | 0.3333 | 0.5055 |
| C | 0.3333 | 0.3333 | 0.5381 |
| C | 0.0000 | 0.0000 | 0.5490 |
| C | 0.0000 | 0.0000 | 0.5816 |
| C | 0.3333 | 0.3333 | 0.5923 |
| F | 0.3333 | 0.3333 | 0.6207 |
| F | 0.0000 | 0.0000 | 0.3793 |
| Tx | 4.7856 | 0.0000 | 0.0000 |
| Ty | 2.3928 | 4.1444 | 0.0000 |
| Tz | 0.0000 | 0.0000 | 90.7069 |

| 6 layers | | | | |
|---|---|---|---|---|
| Atom | X | Y | Z | Bader's Charges |
| C | 0.0000 | 0.0000 | 0.4276 | 0.776 |
| C | 0.3333 | 0.3333 | 0.4382 | -0.016 |
| C | 0.3333 | 0.3333 | 0.4709 | 0.002 |
| C | 0.0000 | 0.0000 | 0.4818 | -0.004 |
| C | 0.0000 | 0.0000 | 0.5144 | 0.003 |
| C | 0.3333 | 0.3333 | 0.5253 | -0.002 |
| C | 0.3333 | 0.3333 | 0.5580 | -0.002 |
| C | 0.0000 | 0.0000 | 0.5689 | 0.003 |
| C | 0.0000 | 0.0000 | 0.6015 | -0.004 |
| C | 0.3333 | 0.3333 | 0.6124 | 0.002 |
| C | 0.3333 | 0.3333 | 0.6451 | -0.016 |
| C | 0.3333 | 0.3333 | 0.6557 | 0.776 |
| F | 0.0000 | 0.0000 | 0.6842 | -0.758 |
| F | 0.0000 | 0.0000 | 0.3991 | -0.758 |
| Tx | 4.7797 | 0.0000 | 0.0000 | |
| Ty | 2.3899 | 4.1393 | 0.0000 | |
| Tz | 0.0000 | 0.0000 | 90.7069 | |

**Table S2.** Information about H-(0001) films: atomic coordinates and lattice vectors.

| 1 layer | | | |
|---|---|---|---|
| Atom | X | Y | Z |
| C | 0.0000 | 0.0000 | 0.4939 |
| C | 0.3333 | 0.3333 | 0.5061 |
| H | 0.3333 | 0.3333 | 0.5356 |
| H | 0.0000 | 0.0000 | 0.4644 |
| Tx | 4.8007 | 0.0000 | 0.0000 |
| Ty | 2.4003 | 4.1575 | 0.0000 |
| Tz | 0.0000 | 0.0000 | 71.0681 |
| 2 layers | | | |

| Atom | X | Y | Z |
|---|---|---|---|
| C | 0.0000 | 0.0000 | 0.4179 |
| C | 0.3333 | 0.3333 | 0.4302 |
| C | 0.3333 | 0.3333 | 0.4698 |
| C | 0.0000 | 0.0000 | 0.4821 |
| H | 0.0000 | 0.0000 | 0.5098 |
| H | 0.0000 | 0.0000 | 0.3902 |
| Tx | 4.7712 | 0.0000 | 0.0000 |
| Ty | 2.3856 | 4.1320 | 0.0000 |
| Tz | 0.0000 | 0.0000 | 75.5890 |
| 3 layers | | | |
| Atom | X | Y | Z |
| C | 0.0000 | 0.0000 | 0.4054 |
| C | 0.3333 | 0.3333 | 0.4166 |
| C | 0.3333 | 0.3333 | 0.4524 |
| C | 0.0000 | 0.0000 | 0.4643 |
| C | 0.0000 | 0.0000 | 0.5001 |
| C | 0.3333 | 0.3333 | 0.5113 |
| H | 0.3333 | 0.3333 | 0.5364 |
| H | 0.0000 | 0.0000 | 0.3802 |
| Tx | 4.7632 | 0.0000 | 0.0000 |
| Ty | 2.3816 | 4.1251 | 0.0000 |
| Tz | 0.0000 | 0.0000 | 83.1479 |
| 4 layers | | | |
| Atom | X | Y | Z |
| C | 0.0000 | 0.0000 | 0.4266 |
| C | 0.3333 | 0.3333 | 0.4373 |
| C | 0.3333 | 0.3333 | 0.4716 |
| C | 0.0000 | 0.0000 | 0.4830 |
| C | 0.0000 | 0.0000 | 0.5170 |
| C | 0.3333 | 0.3333 | 0.5284 |
| C | 0.3333 | 0.3333 | 0.5627 |
| C | 0.0000 | 0.0000 | 0.5734 |
| H | 0.0000 | 0.0000 | 0.5974 |
| H | 0.0000 | 0.0000 | 0.4026 |
| Tx | 4.7594 | 0.0000 | 0.0000 |
| Ty | 2.3797 | 4.1217 | 0.0000 |
| Tz | 0.0000 | 0.0000 | 86.9274 |
| 5 layers | | | |
| Atom | X | Y | Z |
| C | 0.0000 | 0.0000 | 0.4079 |
| C | 0.3333 | 0.3333 | 0.4182 |
| C | 0.3333 | 0.3333 | 0.4510 |
| C | 0.0000 | 0.0000 | 0.4619 |
| C | 0.0000 | 0.0000 | 0.4945 |
| C | 0.3333 | 0.3333 | 0.5055 |
| C | 0.3333 | 0.3333 | 0.5381 |
| C | 0.0000 | 0.0000 | 0.5490 |
| C | 0.0000 | 0.0000 | 0.5818 |
| C | 0.3333 | 0.3333 | 0.5921 |

| | | | | |
|---|---|---|---|---|
| H | 0.3333 | 0.3333 | 0.6151 | |
| H | 0.0000 | 0.0000 | 0.3849 | |
| Tx | 4.7571 | 0.0000 | 0.0000 | |
| Ty | 2.3785 | 4.1197 | 0.0000 | |
| Tz | 0.0000 | 0.0000 | 90.7069 | |
| 6 layers | | | | |
| Atom | X | Y | Z | Bader's Charges |
| C | 0.0000 | 0.0000 | 0.4278 | 0.008 |
| C | 0.3333 | 0.3333 | 0.4381 | 0.001 |
| C | 0.3333 | 0.3333 | 0.4709 | 0.000 |
| C | 0.0000 | 0.0000 | 0.4818 | 0.000 |
| C | 0.0000 | 0.0000 | 0.5144 | 0.000 |
| C | 0.3333 | 0.3333 | 0.5254 | 0.000 |
| C | 0.3333 | 0.3333 | 0.5580 | 0.000 |
| C | 0.0000 | 0.0000 | 0.5689 | 0.000 |
| C | 0.0000 | 0.0000 | 0.6015 | 0.000 |
| C | 0.3333 | 0.3333 | 0.6124 | 0.000 |
| C | 0.3333 | 0.3333 | 0.6453 | 0.001 |
| C | 0.3333 | 0.3333 | 0.6555 | 0.008 |
| H | 0.0000 | 0.0000 | 0.6786 | -0.009 |
| H | 0.0000 | 0.0000 | 0.4047 | -0.009 |
| Tx | 4.7555 | 0.0000 | 0.0000 | |
| Ty | 2.3777 | 4.1183 | 0.0000 | |
| Tz | 0.0000 | 0.0000 | 90.7069 | |

**Table S3.** Information about F-(110) films: atomic coordinates and lattice vectors.

| 1 layer | | | |
|---|---|---|---|
| Atom | X | Y | Z |
| C | 0.0000 | 0.8587 | 0.5135 |
| C | 0.0000 | 0.1413 | 0.4865 |
| C | 0.5000 | 0.3587 | 0.4865 |
| C | 0.5000 | 0.6413 | 0.5135 |
| F | 0.0000 | 0.9938 | 0.5466 |
| F | 0.0000 | 0.0062 | 0.4534 |
| F | 0.5000 | 0.4938 | 0.4534 |
| F | 0.5000 | 0.5062 | 0.5466 |
| Tx | 4.9738 | 0.0000 | 0.0000 |
| Ty | 0.0000 | 7.9212 | 0.0000 |
| Tz | 0.0000 | 0.0000 | 71.5375 |
| 2 layers | | | |
| Atom | X | Y | Z |
| C | 0.2500 | 0.1175 | 0.5000 |
| C | 0.7500 | 0.8825 | 0.5000 |
| C | 0.2500 | 0.3850 | 0.5293 |
| C | 0.7500 | 0.6150 | 0.4707 |
| C | 0.2500 | 0.3850 | 0.4707 |
| C | 0.7500 | 0.6150 | 0.5293 |
| F | 0.2500 | 0.2467 | 0.5603 |

| | | | |
|---|---|---|---|
| F | 0.7500 | 0.7533 | 0.4397 |
| F | 0.2500 | 0.2467 | 0.4397 |
| F | 0.7500 | 0.7533 | 0.5603 |
| Tx | 4.9341 | 0.0000 | 0.0000 |
| Ty | 0.0000 | 7.4836 | 0.0000 |
| Tz | 0.0000 | 0.0000 | 75.5890 |
| 3 layers | | | |
| Atom | X | Y | Z |
| C | 0.0000 | 0.8650 | 0.5428 |
| C | 0.0000 | 0.1350 | 0.4572 |
| C | 0.5000 | 0.3650 | 0.4572 |
| C | 0.5000 | 0.6350 | 0.5428 |
| C | 0.0000 | 0.1293 | 0.5151 |
| C | 0.0000 | 0.8707 | 0.4849 |
| C | 0.5000 | 0.6293 | 0.4849 |
| C | 0.5000 | 0.3707 | 0.5151 |
| F | 0.0000 | 0.9951 | 0.4276 |
| F | 0.0000 | 0.0049 | 0.5724 |
| F | 0.5000 | 0.4951 | 0.5724 |
| F | 0.5000 | 0.5049 | 0.4276 |
| Tx | 4.9035 | 0.0000 | 0.0000 |
| Ty | 0.0000 | 7.3248 | 0.0000 |
| Tz | 0.0000 | 0.0000 | 79.3685 |
| 4 layers | | | |
| Atom | X | Y | Z |
| C | 0.2500 | 0.6154 | 0.4447 |
| C | 0.7500 | 0.3846 | 0.5553 |
| C | 0.2500 | 0.6154 | 0.5553 |
| C | 0.7500 | 0.3846 | 0.4447 |
| C | 0.2500 | 0.8791 | 0.4714 |
| C | 0.7500 | 0.1209 | 0.5286 |
| C | 0.2500 | 0.8791 | 0.5286 |
| C | 0.7500 | 0.1209 | 0.4714 |
| C | 0.2500 | 0.6250 | 0.5000 |
| C | 0.7500 | 0.3750 | 0.5000 |
| F | 0.2500 | 0.7561 | 0.5836 |
| F | 0.7500 | 0.2439 | 0.4164 |
| F | 0.2500 | 0.7561 | 0.4164 |
| F | 0.7500 | 0.2439 | 0.5836 |
| Tx | 4.8832 | 0.0000 | 0.0000 |
| Ty | 0.0000 | 7.2127 | 0.0000 |
| Tz | 0.0000 | 0.0000 | 83.1479 |
| 5 layers | | | |
| Atom | X | Y | Z |
| C | 0.0000 | 0.1291 | 0.5410 |
| C | 0.0000 | 0.8709 | 0.4590 |
| C | 0.5000 | 0.6291 | 0.4590 |
| C | 0.5000 | 0.3709 | 0.5410 |
| C | 0.0000 | 0.8750 | 0.5136 |
| C | 0.0000 | 0.1250 | 0.4864 |

| | | | | |
|---|---|---|---|---|
| C | 0.5000 | 0.3750 | 0.4864 | |
| C | 0.5000 | 0.6250 | 0.5136 | |
| C | 0.0000 | 0.8657 | 0.5667 | |
| C | 0.0000 | 0.1343 | 0.4333 | |
| C | 0.5000 | 0.3657 | 0.4333 | |
| C | 0.5000 | 0.6343 | 0.5667 | |
| F | 0.0000 | 0.9931 | 0.4063 | |
| F | 0.0000 | 0.0069 | 0.5937 | |
| F | 0.5000 | 0.4931 | 0.5937 | |
| F | 0.5000 | 0.5069 | 0.4063 | |
| Tx | 4.8685 | 0.0000 | 0.0000 | |
| Ty | 0.0000 | 7.1394 | 0.0000 | |
| Tz | 0.0000 | 0.0000 | 86.9274 | |
| 6 layers | | | | |
| Atom | X | Y | Z | Bader's Charges |
| C | 0.2500 | 0.6249 | 0.5262 | -0.005 |
| C | 0.7500 | 0.3751 | 0.4738 | -0.005 |
| C | 0.2500 | 0.6249 | 0.4738 | -0.005 |
| C | 0.7500 | 0.3751 | 0.5262 | -0.005 |
| C | 0.2500 | 0.8749 | 0.5000 | 0.004 |
| C | 0.7500 | 0.1251 | 0.5000 | 0.004 |
| C | 0.2500 | 0.8791 | 0.5524 | -0.005 |
| C | 0.7500 | 0.1209 | 0.4476 | -0.005 |
| C | 0.2500 | 0.8791 | 0.4476 | -0.005 |
| C | 0.7500 | 0.1209 | 0.5524 | -0.005 |
| C | 0.2500 | 0.6159 | 0.5771 | 0.802 |
| C | 0.7500 | 0.3841 | 0.4229 | 0.802 |
| C | 0.2500 | 0.6159 | 0.4229 | 0.802 |
| C | 0.7500 | 0.3841 | 0.5771 | 0.802 |
| F | 0.2500 | 0.7573 | 0.3970 | -0.795 |
| F | 0.7500 | 0.2427 | 0.6030 | -0.795 |
| F | 0.2500 | 0.7573 | 0.6030 | -0.795 |
| F | 0.7500 | 0.2427 | 0.3970 | -0.795 |
| Tx | 4.7357 | 0.0000 | 0.0000 | |
| Ty | 0.0000 | 6.7388 | 0.0000 | |
| Tz | 0.0000 | 0.0000 | 90.7069 | |

**Table S4.** Information about H-(110) films: atomic coordinates and lattice vectors.

| 1 layer | | | |
|---|---|---|---|
| Atom | X | Y | Z |
| C | 0.0000 | 0.8639 | 0.5150 |
| C | 0.0000 | 0.1361 | 0.4850 |
| C | 0.5000 | 0.3639 | 0.4850 |
| C | 0.5000 | 0.6361 | 0.5150 |
| H | 0.0000 | 0.0082 | 0.5403 |
| H | 0.0000 | 0.9918 | 0.4597 |
| H | 0.5000 | 0.5082 | 0.4597 |

| | | | |
|---|---|---|---|
| H | 0.5000 | 0.4918 | 0.5403 |
| Tx | 4.8158 | 0.0000 | 0.0000 |
| Ty | 0.0000 | 7.2308 | 0.0000 |
| Tz | 0.0000 | 0.0000 | 71.5375 |
| 2 layers | | | |
| Atom | X | Y | Z |
| C | 0.2500 | 0.1226 | 0.5000 |
| C | 0.7500 | 0.8774 | 0.5000 |
| C | 0.2500 | 0.3817 | 0.5306 |
| C | 0.7500 | 0.6183 | 0.4694 |
| C | 0.2500 | 0.3817 | 0.4694 |
| C | 0.7500 | 0.6183 | 0.5306 |
| H | 0.2500 | 0.2229 | 0.5539 |
| H | 0.7500 | 0.7771 | 0.4461 |
| H | 0.2500 | 0.2229 | 0.4461 |
| H | 0.7500 | 0.7771 | 0.5539 |
| Tx | 4.8045 | 0.0000 | 0.0000 |
| Ty | 0.0000 | 6.9507 | 0.0000 |
| Tz | 0.0000 | 0.0000 | 75.5890 |
| 3 layers | | | |
| Atom | X | Y | Z |
| C | 0.0000 | 0.8685 | 0.5443 |
| C | 0.0000 | 0.1315 | 0.4557 |
| C | 0.5000 | 0.3685 | 0.4557 |
| C | 0.5000 | 0.6315 | 0.5443 |
| C | 0.0000 | 0.1260 | 0.5151 |
| C | 0.0000 | 0.8740 | 0.4849 |
| C | 0.5000 | 0.6260 | 0.4849 |
| C | 0.5000 | 0.3740 | 0.5151 |
| H | 0.0000 | 0.9699 | 0.4336 |
| H | 0.0000 | 0.0301 | 0.5664 |
| H | 0.5000 | 0.4699 | 0.5664 |
| H | 0.5000 | 0.5301 | 0.4336 |
| Tx | 4.7984 | 0.0000 | 0.0000 |
| Ty | 0.0000 | 6.8891 | 0.0000 |
| Tz | 0.0000 | 0.0000 | 79.3685 |
| 4 layers | | | |
| Atom | X | Y | Z |
| C | 0.2500 | 0.6188 | 0.4434 |
| C | 0.7500 | 0.3812 | 0.5566 |
| C | 0.2500 | 0.6188 | 0.5566 |
| C | 0.7500 | 0.3812 | 0.4434 |
| C | 0.2500 | 0.8761 | 0.4713 |
| C | 0.7500 | 0.1239 | 0.5287 |
| C | 0.2500 | 0.8761 | 0.5287 |
| C | 0.7500 | 0.1239 | 0.4713 |
| C | 0.2500 | 0.6254 | 0.5000 |
| C | 0.7500 | 0.3746 | 0.5000 |
| H | 0.2500 | 0.7819 | 0.5777 |
| H | 0.7500 | 0.2181 | 0.4223 |

| | | | | |
|---|---|---|---|---|
| H | 0.2500 | 0.7819 | 0.4223 | |
| H | 0.7500 | 0.2181 | 0.5777 | |
| Tx | 4.7943 | 0.0000 | 0.0000 | |
| Ty | 0.0000 | 6.8549 | 0.0000 | |
| Tz | 0.0000 | 0.0000 | 83.1479 | |
| 5 layers | | | | |
| Atom | X | Y | Z | |
| C | 0.0000 | 0.1260 | 0.5412 | |
| C | 0.0000 | 0.8740 | 0.4588 | |
| C | 0.5000 | 0.6260 | 0.4588 | |
| C | 0.5000 | 0.3740 | 0.5412 | |
| C | 0.0000 | 0.8752 | 0.5137 | |
| C | 0.0000 | 0.1248 | 0.4863 | |
| C | 0.5000 | 0.3752 | 0.4863 | |
| C | 0.5000 | 0.6248 | 0.5137 | |
| C | 0.0000 | 0.8689 | 0.5679 | |
| C | 0.0000 | 0.1311 | 0.4321 | |
| C | 0.5000 | 0.3689 | 0.4321 | |
| C | 0.5000 | 0.6311 | 0.5679 | |
| H | 0.0000 | 0.9671 | 0.4120 | |
| H | 0.0000 | 0.0329 | 0.5880 | |
| H | 0.5000 | 0.4671 | 0.5880 | |
| H | 0.5000 | 0.5329 | 0.4120 | |
| Tx | 4.7914 | 0.0000 | 0.0000 | |
| Ty | 0.0000 | 6.8347 | 0.0000 | |
| Tz | 0.0000 | 0.0000 | 86.9274 | |
| 6 layers | | | | |
| Atom | X | Y | Z | Bader's Charges |
| C | 0.2500 | 0.6252 | 0.5263 | -0.001 |
| C | 0.7500 | 0.3748 | 0.4737 | -0.001 |
| C | 0.2500 | 0.6252 | 0.4737 | -0.001 |
| C | 0.7500 | 0.3748 | 0.5263 | -0.001 |
| C | 0.2500 | 0.8750 | 0.5000 | 0.001 |
| C | 0.7500 | 0.1250 | 0.5000 | 0.001 |
| C | 0.2500 | 0.8760 | 0.5526 | 0.001 |
| C | 0.7500 | 0.1240 | 0.4474 | 0.001 |
| C | 0.2500 | 0.8760 | 0.4474 | 0.001 |
| C | 0.7500 | 0.1240 | 0.5526 | 0.001 |
| C | 0.2500 | 0.6191 | 0.5783 | -0.002 |
| C | 0.7500 | 0.3809 | 0.4217 | -0.002 |
| C | 0.2500 | 0.6191 | 0.4217 | -0.002 |
| C | 0.7500 | 0.3809 | 0.5783 | -0.002 |
| H | 0.2500 | 0.7836 | 0.4025 | 0.002 |
| H | 0.7500 | 0.2164 | 0.5975 | 0.002 |
| H | 0.2500 | 0.7836 | 0.5975 | 0.002 |
| H | 0.7500 | 0.2164 | 0.4025 | 0.002 |
| Tx | 4.7894 | 0.0000 | 0.0000 | |
| Ty | 0.0000 | 6.8207 | 0.0000 | |
| Tz | 0.0000 | 0.0000 | 90.7069 | |

**Table S5.** Information about F-(111) films: atomic coordinates and lattice vectors.

| 1 layer | | | |
|---|---|---|---|
| Atom | X | Y | Z |
| C | 0.0000 | 0.0000 | 0.4934 |
| C | 0.3333 | 0.6667 | 0.5066 |
| F | 0.3333 | 0.6667 | 0.5431 |
| F | 0.0000 | 0.0000 | 0.4569 |
| Tx | 4.9266 | 0.0000 | 0.0000 |
| Ty | -2.4633 | 4.2665 | 0.0000 |
| Tz | 0.0000 | 0.0000 | 70.8903 |
| 2 layers | | | |
| Atom | X | Y | Z |
| C | 0.0000 | 0.0000 | 0.4777 |
| C | 0.6667 | 0.3333 | 0.4904 |
| C | 0.0000 | 0.0000 | 0.4389 |
| C | 0.3333 | 0.6667 | 0.4263 |
| F | 0.3333 | 0.6667 | 0.3921 |
| F | 0.6667 | 0.3333 | 0.5246 |
| Tx | 4.8483 | 0.0000 | 0.0000 |
| Ty | -2.4242 | 4.1988 | 0.0000 |
| Tz | 0.0000 | 0.0000 | 75.5890 |
| 3 layers | | | |
| Atom | X | Y | Z |
| C | 0.0000 | 0.0000 | 0.4172 |
| C | 0.3333 | 0.6667 | 0.4058 |
| C | 0.0000 | 0.0000 | 0.4525 |
| C | 0.6667 | 0.3333 | 0.4642 |
| C | 0.6667 | 0.3333 | 0.4994 |
| C | 0.3333 | 0.6667 | 0.5109 |
| F | 0.3333 | 0.6667 | 0.5420 |
| F | 0.3333 | 0.6667 | 0.3747 |
| Tx | 4.8237 | 0.0000 | 0.0000 |
| Ty | -2.4119 | 4.1775 | 0.0000 |
| Tz | 0.0000 | 0.0000 | 83.1479 |
| 4 layers | | | |
| Atom | X | Y | Z |
| C | 0.6667 | 0.3333 | 0.3966 |
| C | 0.6667 | 0.3333 | 0.5310 |
| C | 0.0000 | 0.0000 | 0.3856 |
| C | 0.0000 | 0.0000 | 0.5201 |
| C | 0.6667 | 0.3333 | 0.4303 |
| C | 0.3333 | 0.6667 | 0.4415 |
| C | 0.3333 | 0.6667 | 0.4751 |
| C | 0.0000 | 0.0000 | 0.4863 |
| F | 0.6667 | 0.3333 | 0.5608 |
| F | 0.0000 | 0.0000 | 0.3559 |
| Tx | 4.8117 | 0.0000 | 0.0000 |

| | | | | |
|---|---|---|---|---|
| Ty | -2.4058 | 4.1670 | 0.0000 | |
| Tz | 0.0000 | 0.0000 | 86.9274 | |
| 5 layers | | | | |
| Atom | X | Y | Z | |
| C | 0.0000 | 0.0000 | 0.4085 | |
| C | 0.6667 | 0.3333 | 0.4190 | |
| C | 0.6667 | 0.3333 | 0.4515 | |
| C | 0.3333 | 0.6667 | 0.4623 | |
| C | 0.3333 | 0.6667 | 0.4946 | |
| C | 0.0000 | 0.0000 | 0.5054 | |
| C | 0.0000 | 0.0000 | 0.5377 | |
| C | 0.6667 | 0.3333 | 0.5485 | |
| C | 0.6667 | 0.3333 | 0.5810 | |
| C | 0.3333 | 0.6667 | 0.5915 | |
| F | 0.3333 | 0.6667 | 0.6198 | |
| F | 0.0000 | 0.0000 | 0.3802 | |
| Tx | 4.7702 | 0.0000 | 0.0000 | |
| Ty | -2.3851 | 4.1311 | 0.0000 | |
| Tz | 0.0000 | 0.0000 | 90.7069 | |
| 6 layers | | | | |
| Atom | X | Y | Z | Bader's Charges |
| C | 0.0000 | 0.0000 | 0.6081 | 0.755 |
| C | 0.0000 | 0.0000 | 0.3919 | 0.755 |
| C | 0.3333 | 0.6667 | 0.4020 | -0.005 |
| C | 0.6667 | 0.3333 | 0.5980 | -0.005 |
| C | 0.3333 | 0.6667 | 0.4330 | 0.002 |
| C | 0.6667 | 0.3333 | 0.5670 | 0.002 |
| C | 0.3333 | 0.6667 | 0.5567 | -0.005 |
| C | 0.6667 | 0.3333 | 0.4433 | -0.005 |
| C | 0.3333 | 0.6667 | 0.5258 | 0.003 |
| C | 0.6667 | 0.3333 | 0.4742 | 0.003 |
| C | 0.0000 | 0.0000 | 0.5155 | -0.002 |
| C | 0.0000 | 0.0000 | 0.4845 | -0.002 |
| F | 0.0000 | 0.0000 | 0.3645 | -0.749 |
| F | 0.0000 | 0.0000 | 0.6355 | -0.749 |
| Tx | 4.7996 | 0.0000 | 0.0000 | |
| Ty | -2.3998 | 4.1566 | 0.0000 | |
| Tz | 0.0000 | 0.0000 | 94.4863 | |

**Table S6.** Information about H-(111) films: atomic coordinates and lattice vectors.

| 1 layer | | | |
|---|---|---|---|
| Atom | X | Y | Z |
| C | 0.0000 | 0.0000 | 0.4939 |
| C | 0.3333 | 0.6667 | 0.5061 |
| H | 0.3333 | 0.6667 | 0.5357 |
| H | 0.0000 | 0.0000 | 0.4643 |
| Tx | 4.8008 | 0.0000 | 0.0000 |
| Ty | -2.4004 | 4.1576 | 0.0000 |

| | | | |
|---|---|---|---|
| Tz | 0.0000 | 0.0000 | 70.8903 |
| 2 layers | | | |
| Atom | X | Y | Z |
| C | 0.0000 | 0.0000 | 0.4778 |
| C | 0.6667 | 0.3333 | 0.4900 |
| C | 0.0000 | 0.0000 | 0.4388 |
| C | 0.3333 | 0.6667 | 0.4267 |
| H | 0.3333 | 0.6667 | 0.3990 |
| H | 0.6667 | 0.3333 | 0.5177 |
| Tx | 4.7843 | 0.0000 | 0.0000 |
| Ty | -2.3922 | 4.1434 | 0.0000 |
| Tz | 0.0000 | 0.0000 | 75.5890 |
| 3 layers | | | |
| Atom | X | Y | Z |
| C | 0.0000 | 0.0000 | 0.4172 |
| C | 0.3333 | 0.6667 | 0.4061 |
| C | 0.0000 | 0.0000 | 0.4525 |
| C | 0.6667 | 0.3333 | 0.4642 |
| C | 0.6667 | 0.3333 | 0.4995 |
| C | 0.3333 | 0.6667 | 0.5106 |
| H | 0.3333 | 0.6667 | 0.5357 |
| H | 0.3333 | 0.6667 | 0.3809 |
| Tx | 4.7805 | 0.0000 | 0.0000 |
| Ty | -2.3902 | 4.1400 | 0.0000 |
| Tz | 0.0000 | 0.0000 | 83.1479 |
| 4 layers | | | |
| Atom | X | Y | Z |
| C | 0.6667 | 0.3333 | 0.3966 |
| C | 0.6667 | 0.3333 | 0.5307 |
| C | 0.0000 | 0.0000 | 0.3860 |
| C | 0.0000 | 0.0000 | 0.5201 |
| C | 0.6667 | 0.3333 | 0.4303 |
| C | 0.3333 | 0.6667 | 0.4415 |
| C | 0.3333 | 0.6667 | 0.4752 |
| C | 0.0000 | 0.0000 | 0.4863 |
| H | 0.6667 | 0.3333 | 0.5548 |
| H | 0.0000 | 0.0000 | 0.3619 |
| Tx | 4.7789 | 0.0000 | 0.0000 |
| Ty | -2.3894 | 4.1386 | 0.0000 |
| Tz | 0.0000 | 0.0000 | 86.9274 |
| 5 layers | | | |
| Atom | X | Y | Z |
| C | 0.0000 | 0.0000 | 0.4092 |
| C | 0.6667 | 0.3333 | 0.4193 |
| C | 0.6667 | 0.3333 | 0.4517 |
| C | 0.3333 | 0.6667 | 0.4624 |
| C | 0.3333 | 0.6667 | 0.4946 |
| C | 0.0000 | 0.0000 | 0.5054 |
| C | 0.0000 | 0.0000 | 0.5376 |
| C | 0.6667 | 0.3333 | 0.5483 |

| | | | |
|---|---|---|---|
| C | 0.6667 | 0.3333 | 0.5807 |
| C | 0.3333 | 0.6667 | 0.5908 |
| H | 0.3333 | 0.6667 | 0.6139 |
| H | 0.0000 | 0.0000 | 0.3861 |
| Tx | 4.7780 | 0.0000 | 0.0000 |
| Ty | -2.3890 | 4.1378 | 0.0000 |
| Tz | 0.0000 | 0.0000 | 90.7069 |
| 6 layers | | | |
| Atom | X | Y | Z |
| C | 0.0000 | 0.0000 | 0.6078 |
| C | 0.0000 | 0.0000 | 0.3922 |
| C | 0.3333 | 0.6667 | 0.4019 |
| C | 0.6667 | 0.3333 | 0.5981 |
| C | 0.3333 | 0.6667 | 0.4330 |
| C | 0.6667 | 0.3333 | 0.5670 |
| C | 0.3333 | 0.6667 | 0.5567 |
| C | 0.6667 | 0.3333 | 0.4433 |
| C | 0.3333 | 0.6667 | 0.5258 |
| C | 0.6667 | 0.3333 | 0.4742 |
| C | 0.0000 | 0.0000 | 0.5155 |
| C | 0.0000 | 0.0000 | 0.4845 |
| H | 0.0000 | 0.0000 | 0.3700 |
| H | 0.0000 | 0.0000 | 0.6300 |
| Tx | 4.7774 | 0.0000 | 0.0000 |
| Ty | -2.3887 | 4.1373 | 0.0000 |
| Tz | 0.0000 | 0.0000 | 94.4863 |

**Table S7.** Information about F-$(10\bar{1}0)$ films: atomic coordinates and lattice vectors.

| 1 layer | | | |
|---|---|---|---|
| Atom | X | Y | Z |
| C | 0.4307 | 0.2500 | 0.5085 |
| C | 0.5693 | 0.7500 | 0.4915 |
| C | 0.0693 | 0.2500 | 0.5085 |
| C | 0.9307 | 0.7500 | 0.4915 |
| F | 0.0043 | 0.2500 | 0.5442 |
| F | 0.9957 | 0.7500 | 0.4558 |
| F | 0.4957 | 0.2500 | 0.5442 |
| F | 0.5043 | 0.7500 | 0.4558 |
| Tx | 8.7141 | 0.0000 | 0.0000 |
| Ty | 0.0000 | 4.8783 | 0.0000 |
| Tz | 0.0000 | 0.0000 | 70.5454 |
| 2 layers | | | |
| Atom | X | Y | Z |
| C | 0.5668 | 0.5000 | 0.5353 |
| C | 0.4332 | 0.5000 | 0.4647 |
| C | 0.9332 | 0.5000 | 0.5353 |
| C | 0.0668 | 0.5000 | 0.4647 |
| C | 0.4362 | 0.0000 | 0.5182 |

| | | | |
|---|---|---|---|
| C | 0.5638 | 0.0000 | 0.4818 |
| C | 0.0638 | 0.0000 | 0.5182 |
| C | 0.9362 | 0.0000 | 0.4818 |
| F | 0.0008 | 0.5000 | 0.4315 |
| F | 0.9992 | 0.5000 | 0.5685 |
| F | 0.4992 | 0.5000 | 0.4315 |
| F | 0.5008 | 0.5000 | 0.5685 |
| Tx | 8.3556 | 0.0000 | 0.0000 |
| Ty | 0.0000 | 4.8068 | 0.0000 |
| Tz | 0.0000 | 0.0000 | 75.5890 |
| 3 layers | | | |
| Atom | X | Y | Z |
| C | 0.2500 | 0.0663 | 0.5581 |
| C | 0.7500 | 0.9337 | 0.4419 |
| C | 0.2500 | 0.4337 | 0.5581 |
| C | 0.7500 | 0.5663 | 0.4419 |
| C | 0.2500 | 0.0643 | 0.4578 |
| C | 0.7500 | 0.9357 | 0.5422 |
| C | 0.2500 | 0.4357 | 0.4578 |
| C | 0.7500 | 0.5643 | 0.5422 |
| C | 0.2500 | 0.9387 | 0.4916 |
| C | 0.7500 | 0.0613 | 0.5084 |
| C | 0.2500 | 0.5613 | 0.4916 |
| C | 0.7500 | 0.4387 | 0.5084 |
| F | 0.2500 | 0.4999 | 0.5889 |
| F | 0.7500 | 0.5001 | 0.4111 |
| F | 0.2500 | 0.0001 | 0.5889 |
| F | 0.7500 | 0.9999 | 0.4111 |
| Tx | 4.8066 | 0.0000 | 0.0000 |
| Ty | 0.0000 | 8.2152 | 0.0000 |
| Tz | 0.0000 | 0.0000 | 81.2582 |
| 4 layers | | | |
| Atom | X | Y | Z |
| C | 0.9336 | 0.0000 | 0.5781 |
| C | 0.0664 | 0.0000 | 0.4219 |
| C | 0.5664 | 0.0000 | 0.5781 |
| C | 0.4336 | 0.0000 | 0.4219 |
| C | 0.0649 | 0.5000 | 0.5632 |
| C | 0.9351 | 0.5000 | 0.4368 |
| C | 0.4351 | 0.5000 | 0.5632 |
| C | 0.5649 | 0.5000 | 0.4368 |
| C | 0.9380 | 0.5000 | 0.5315 |
| C | 0.0620 | 0.5000 | 0.4685 |
| C | 0.5620 | 0.5000 | 0.5315 |
| C | 0.4380 | 0.5000 | 0.4685 |
| C | 0.0624 | 0.0000 | 0.5158 |
| C | 0.9376 | 0.0000 | 0.4842 |
| C | 0.4376 | 0.0000 | 0.5158 |
| C | 0.5624 | 0.0000 | 0.4842 |
| F | 0.5003 | 0.0000 | 0.3931 |

| | | | | |
|---|---|---|---|---|
| F | 0.4997 | 0.0000 | 0.6069 | |
| F | 0.9997 | 0.0000 | 0.3931 | |
| F | 0.0003 | 0.0000 | 0.6069 | |
| Tx | 8.1437 | 0.0000 | 0.0000 | |
| Ty | 0.0000 | 4.7784 | 0.0000 | |
| Tz | 0.0000 | 0.0000 | 86.9274 | |
| 5 layers | | | | |
| Atom | X | Y | Z | |
| C | 0.4378 | 0.2500 | 0.5507 | |
| C | 0.5622 | 0.7500 | 0.4493 | |
| C | 0.0622 | 0.2500 | 0.5507 | |
| C | 0.9378 | 0.7500 | 0.4493 | |
| C | 0.0625 | 0.2500 | 0.4637 | |
| C | 0.9375 | 0.7500 | 0.5363 | |
| C | 0.4375 | 0.2500 | 0.4637 | |
| C | 0.5625 | 0.7500 | 0.5363 | |
| C | 0.9375 | 0.2500 | 0.4928 | |
| C | 0.0625 | 0.7500 | 0.5072 | |
| C | 0.5625 | 0.2500 | 0.4928 | |
| C | 0.4375 | 0.7500 | 0.5072 | |
| C | 0.9350 | 0.2500 | 0.5799 | |
| C | 0.0650 | 0.7500 | 0.4201 | |
| C | 0.5650 | 0.2500 | 0.5799 | |
| C | 0.4350 | 0.7500 | 0.4201 | |
| C | 0.5663 | 0.2500 | 0.4064 | |
| C | 0.4337 | 0.7500 | 0.5936 | |
| C | 0.9337 | 0.2500 | 0.4064 | |
| C | 0.0663 | 0.7500 | 0.5936 | |
| F | 0.4995 | 0.2500 | 0.3799 | |
| F | 0.5005 | 0.7500 | 0.6201 | |
| F | 0.0005 | 0.2500 | 0.3799 | |
| F | 0.9995 | 0.7500 | 0.6201 | |
| Tx | 8.0978 | 0.0000 | 0.0000 | |
| Ty | 0.0000 | 4.7726 | 0.0000 | |
| Tz | 0.0000 | 0.0000 | 94.4863 | |
| 6 layers | | | | |
| Atom | X | Y | Z | Bader's Charges |
| C | 0.9374 | 0.0000 | 0.5279 | 0.003 |
| C | 0.0626 | 0.0000 | 0.4721 | 0.003 |
| C | 0.5626 | 0.0000 | 0.5279 | 0.003 |
| C | 0.4374 | 0.0000 | 0.4721 | 0.003 |
| C | 0.0626 | 0.5000 | 0.5139 | -0.004 |
| C | 0.9374 | 0.5000 | 0.4861 | -0.004 |
| C | 0.4374 | 0.5000 | 0.5139 | -0.004 |
| C | 0.5626 | 0.5000 | 0.4861 | -0.004 |
| C | 0.0622 | 0.5000 | 0.4303 | 0.001 |
| C | 0.9378 | 0.5000 | 0.5697 | 0.001 |
| C | 0.4378 | 0.5000 | 0.4303 | 0.001 |
| C | 0.5622 | 0.5000 | 0.5697 | 0.001 |
| C | 0.9374 | 0.0000 | 0.4442 | -0.005 |

| | | | | |
|---|---|---|---|---|
| C | 0.0626 | 0.0000 | 0.5558 | -0.005 |
| C | 0.5626 | 0.0000 | 0.4442 | -0.005 |
| C | 0.4374 | 0.0000 | 0.5558 | -0.005 |
| C | 0.9350 | 0.5000 | 0.4023 | 0.007 |
| C | 0.0650 | 0.5000 | 0.5977 | 0.007 |
| C | 0.5650 | 0.5000 | 0.4023 | 0.007 |
| C | 0.4350 | 0.5000 | 0.5977 | 0.007 |
| C | 0.0663 | 0.0000 | 0.3891 | 0.775 |
| C | 0.9337 | 0.0000 | 0.6109 | 0.775 |
| C | 0.4337 | 0.0000 | 0.3891 | 0.775 |
| C | 0.5663 | 0.0000 | 0.6109 | 0.775 |
| F | 0.5006 | 0.0000 | 0.3636 | -0.778 |
| F | 0.4994 | 0.0000 | 0.6364 | -0.778 |
| F | 0.9994 | 0.0000 | 0.3636 | -0.778 |
| F | 0.0006 | 0.0000 | 0.6364 | -0.778 |
| Tx | 8.0667 | 0.0000 | 0.0000 | |
| Ty | 0.0000 | 4.7688 | 0.0000 | |
| Tz | 0.0000 | 0.0000 | 98.2658 | |

**Table S8.** Information about H-($10\bar{1}0$) films: atomic coordinates and lattice vectors.

| 1 layer | | | |
|---|---|---|---|
| Atom | X | Y | Z |
| C | 0.4322 | 0.2500 | 0.5087 |
| C | 0.5678 | 0.7500 | 0.4913 |
| C | 0.0678 | 0.2500 | 0.5087 |
| C | 0.9322 | 0.7500 | 0.4913 |
| H | 0.9944 | 0.2500 | 0.5370 |
| H | 0.0056 | 0.7500 | 0.4630 |
| H | 0.5056 | 0.2500 | 0.5370 |
| H | 0.4944 | 0.7500 | 0.4630 |
| Tx | 8.1467 | 0.0000 | 0.0000 |
| Ty | 0.0000 | 4.7771 | 0.0000 |
| Tz | 0.0000 | 0.0000 | 70.5454 |
| 2 layers | | | |
| Atom | X | Y | Z |
| C | 0.5663 | 0.5000 | 0.5356 |
| C | 0.4337 | 0.5000 | 0.4644 |
| C | 0.9337 | 0.5000 | 0.5356 |
| C | 0.0663 | 0.5000 | 0.4644 |
| C | 0.4371 | 0.0000 | 0.5183 |
| C | 0.5629 | 0.0000 | 0.4817 |
| C | 0.0629 | 0.0000 | 0.5183 |
| C | 0.9371 | 0.0000 | 0.4817 |
| H | 0.9867 | 0.5000 | 0.4382 |
| H | 0.0133 | 0.5000 | 0.5618 |
| H | 0.5133 | 0.5000 | 0.4382 |
| H | 0.4867 | 0.5000 | 0.5618 |
| Tx | 8.0052 | 0.0000 | 0.0000 |

| | | | |
|---|---|---|---|
| Ty | 0.0000 | 4.7601 | 0.0000 |
| Tz | 0.0000 | 0.0000 | 75.5890 |
| 3 layers | | | |
| Atom | X | Y | Z |
| C | 0.2500 | 0.0658 | 0.5583 |
| C | 0.7500 | 0.9342 | 0.4417 |
| C | 0.2500 | 0.4342 | 0.5583 |
| C | 0.7500 | 0.5658 | 0.4417 |
| C | 0.2500 | 0.0630 | 0.4578 |
| C | 0.7500 | 0.9370 | 0.5422 |
| C | 0.2500 | 0.4370 | 0.4578 |
| C | 0.7500 | 0.5630 | 0.5422 |
| C | 0.2500 | 0.9378 | 0.4916 |
| C | 0.7500 | 0.0622 | 0.5084 |
| C | 0.2500 | 0.5622 | 0.4916 |
| C | 0.7500 | 0.4378 | 0.5084 |
| H | 0.2500 | 0.5135 | 0.5827 |
| H | 0.7500 | 0.4865 | 0.4173 |
| H | 0.2500 | 0.9865 | 0.5827 |
| H | 0.7500 | 0.0135 | 0.4173 |
| Tx | 4.7752 | 0.0000 | 0.0000 |
| Ty | 0.0000 | 7.9642 | 0.0000 |
| Tz | 0.0000 | 0.0000 | 81.2582 |
| 4 layers | | | |
| Atom | X | Y | Z |
| C | 0.9341 | 0.0000 | 0.5783 |
| C | 0.0659 | 0.0000 | 0.4217 |
| C | 0.5659 | 0.0000 | 0.5783 |
| C | 0.4341 | 0.0000 | 0.4217 |
| C | 0.0632 | 0.5000 | 0.5632 |
| C | 0.9368 | 0.5000 | 0.4368 |
| C | 0.4368 | 0.5000 | 0.5632 |
| C | 0.5632 | 0.5000 | 0.4368 |
| C | 0.9376 | 0.5000 | 0.5315 |
| C | 0.0624 | 0.5000 | 0.4685 |
| C | 0.5624 | 0.5000 | 0.5315 |
| C | 0.4376 | 0.5000 | 0.4685 |
| C | 0.0627 | 0.0000 | 0.5158 |
| C | 0.9373 | 0.0000 | 0.4842 |
| C | 0.4373 | 0.0000 | 0.5158 |
| C | 0.5627 | 0.0000 | 0.4842 |
| H | 0.5148 | 0.0000 | 0.3990 |
| H | 0.4852 | 0.0000 | 0.6010 |
| H | 0.9852 | 0.0000 | 0.3990 |
| H | 0.0148 | 0.0000 | 0.6010 |
| Tx | 7.9516 | 0.0000 | 0.0000 |
| Ty | 0.0000 | 4.7541 | 0.0000 |
| Tz | 0.0000 | 0.0000 | 86.9274 |
| 5 layers | | | |
| Atom | X | Y | Z |

| | | | | |
|---|---|---|---|---|
| C | 0.4375 | 0.2500 | 0.5508 | |
| C | 0.5625 | 0.7500 | 0.4492 | |
| C | 0.0625 | 0.2500 | 0.5508 | |
| C | 0.9375 | 0.7500 | 0.4492 | |
| C | 0.0627 | 0.2500 | 0.4637 | |
| C | 0.9373 | 0.7500 | 0.5363 | |
| C | 0.4373 | 0.2500 | 0.4637 | |
| C | 0.5627 | 0.7500 | 0.5363 | |
| C | 0.9373 | 0.2500 | 0.4927 | |
| C | 0.0627 | 0.7500 | 0.5073 | |
| C | 0.5627 | 0.2500 | 0.4927 | |
| C | 0.4373 | 0.7500 | 0.5073 | |
| C | 0.9368 | 0.2500 | 0.5799 | |
| C | 0.0632 | 0.7500 | 0.4201 | |
| C | 0.5632 | 0.2500 | 0.5799 | |
| C | 0.4368 | 0.7500 | 0.4201 | |
| C | 0.5659 | 0.2500 | 0.4062 | |
| C | 0.4341 | 0.7500 | 0.5938 | |
| C | 0.9341 | 0.2500 | 0.4062 | |
| C | 0.0659 | 0.7500 | 0.5938 | |
| H | 0.4850 | 0.2500 | 0.3853 | |
| H | 0.5150 | 0.7500 | 0.6147 | |
| H | 0.0150 | 0.2500 | 0.3853 | |
| H | 0.9850 | 0.7500 | 0.6147 | |
| Tx | 7.9410 | 0.0000 | 0.0000 | |
| Ty | 0.0000 | 4.7530 | 0.0000 | |
| Tz | 0.0000 | 0.0000 | 94.4863 | |
| 6 layers | | | | |
| Atom | X | Y | Z | Bader's Charges |
| C | 0.9373 | 0.0000 | 0.5279 | 0.003 |
| C | 0.0627 | 0.0000 | 0.4721 | 0.003 |
| C | 0.5627 | 0.0000 | 0.5279 | 0.003 |
| C | 0.4373 | 0.0000 | 0.4721 | 0.003 |
| C | 0.0627 | 0.5000 | 0.5139 | -0.004 |
| C | 0.9373 | 0.5000 | 0.4861 | -0.004 |
| C | 0.4373 | 0.5000 | 0.5139 | -0.004 |
| C | 0.5627 | 0.5000 | 0.4861 | -0.004 |
| C | 0.0625 | 0.5000 | 0.4303 | 0.004 |
| C | 0.9375 | 0.5000 | 0.5697 | 0.004 |
| C | 0.4375 | 0.5000 | 0.4303 | 0.004 |
| C | 0.5625 | 0.5000 | 0.5697 | 0.004 |
| C | 0.9373 | 0.0000 | 0.4442 | -0.007 |
| C | 0.0627 | 0.0000 | 0.5558 | -0.007 |
| C | 0.5627 | 0.0000 | 0.4442 | -0.007 |
| C | 0.4373 | 0.0000 | 0.5558 | -0.007 |
| C | 0.9368 | 0.5000 | 0.4023 | 0.018 |
| C | 0.0632 | 0.5000 | 0.5977 | 0.018 |
| C | 0.5632 | 0.5000 | 0.4023 | 0.018 |
| C | 0.4368 | 0.5000 | 0.5977 | 0.018 |
| C | 0.0658 | 0.0000 | 0.3889 | -0.050 |

| | | | | |
|---|---|---|---|---|
| C | 0.9342 | 0.0000 | 0.6111 | -0.050 |
| C | 0.4342 | 0.0000 | 0.3889 | -0.050 |
| C | 0.5658 | 0.0000 | 0.6111 | -0.050 |
| H | 0.5152 | 0.0000 | 0.3688 | 0.034 |
| H | 0.4848 | 0.0000 | 0.6312 | 0.034 |
| H | 0.9848 | 0.0000 | 0.3688 | 0.034 |
| H | 0.0152 | 0.0000 | 0.6312 | 0.034 |
| Tx | 7.9343 | 0.0000 | 0.0000 | |
| Ty | 0.0000 | 4.7522 | 0.0000 | |
| Tz | 0.0000 | 0.0000 | 98.2658 | |

**Table S9.** Information about F-($\bar{2}110$) films: atomic coordinates and lattice vectors.

| 1 layer | | | |
|---|---|---|---|
| Atom | X | Y | Z |
| C | 0.9303 | 0.8990 | 0.5137 |
| C | 0.0697 | 0.1010 | 0.4863 |
| C | 0.4303 | 0.1010 | 0.4863 |
| C | 0.5697 | 0.8990 | 0.5137 |
| C | 0.5697 | 0.3990 | 0.4863 |
| C | 0.4303 | 0.6010 | 0.5137 |
| C | 0.0697 | 0.6010 | 0.5137 |
| C | 0.9303 | 0.3990 | 0.4863 |
| F | 0.5057 | 0.4965 | 0.4531 |
| F | 0.4943 | 0.5035 | 0.5469 |
| F | 0.0057 | 0.5035 | 0.5469 |
| F | 0.9943 | 0.4965 | 0.4531 |
| F | 0.9943 | 0.9965 | 0.5469 |
| F | 0.0057 | 0.0035 | 0.4531 |
| F | 0.4943 | 0.0035 | 0.4531 |
| F | 0.5057 | 0.9965 | 0.5469 |
| Tx | 8.7281 | 0.0000 | 0.0000 |
| Ty | 0.0000 | 9.2558 | 0.0000 |
| Tz | 0.0000 | 0.0000 | 70.7627 |
| 2 layers | | | |
| Atom | X | Y | Z |
| C | 0.4368 | 0.9136 | 0.4842 |
| C | 0.5632 | 0.0864 | 0.5158 |
| C | 0.9368 | 0.0864 | 0.5158 |
| C | 0.0632 | 0.9136 | 0.4842 |
| C | 0.0632 | 0.4136 | 0.5158 |
| C | 0.9368 | 0.5864 | 0.4842 |
| C | 0.5632 | 0.5864 | 0.4842 |
| C | 0.4368 | 0.4136 | 0.5158 |
| C | 0.4318 | 0.9049 | 0.5448 |
| C | 0.5682 | 0.0951 | 0.4552 |
| C | 0.9318 | 0.0951 | 0.4552 |
| C | 0.0682 | 0.9049 | 0.5448 |
| C | 0.0682 | 0.4049 | 0.4552 |

| | | | |
|---|---|---|---|
| C | 0.9318 | 0.5951 | 0.5448 |
| C | 0.5682 | 0.5951 | 0.5448 |
| C | 0.4318 | 0.4049 | 0.4552 |
| F | 0.0015 | 0.5052 | 0.4243 |
| F | 0.9985 | 0.4948 | 0.5757 |
| F | 0.5015 | 0.4948 | 0.5757 |
| F | 0.4985 | 0.5052 | 0.4243 |
| F | 0.4985 | 0.0052 | 0.5757 |
| F | 0.5015 | 0.9948 | 0.4243 |
| F | 0.9985 | 0.9948 | 0.4243 |
| F | 0.0015 | 0.0052 | 0.5757 |
| Tx | 8.3641 | 0.0000 | 0.0000 |
| Ty | 0.0000 | 8.7492 | 0.0000 |
| Tz | 0.0000 | 0.0000 | 75.5890 |
| 3 layers | | | |
| Atom | X | Y | Z |
| C | 0.4320 | 0.4060 | 0.4290 |
| C | 0.5680 | 0.5940 | 0.5710 |
| C | 0.9320 | 0.5940 | 0.5710 |
| C | 0.0680 | 0.4060 | 0.4290 |
| C | 0.0680 | 0.9060 | 0.5710 |
| C | 0.9320 | 0.0940 | 0.4290 |
| C | 0.5680 | 0.0940 | 0.4290 |
| C | 0.4320 | 0.9060 | 0.5710 |
| C | 0.4381 | 0.4171 | 0.4854 |
| C | 0.5619 | 0.5829 | 0.5146 |
| C | 0.9381 | 0.5829 | 0.5146 |
| C | 0.0619 | 0.4171 | 0.4854 |
| C | 0.0619 | 0.9171 | 0.5146 |
| C | 0.9381 | 0.0829 | 0.4854 |
| C | 0.5619 | 0.0829 | 0.4854 |
| C | 0.4381 | 0.9171 | 0.5146 |
| C | 0.4361 | 0.4130 | 0.5438 |
| C | 0.5639 | 0.5870 | 0.4562 |
| C | 0.9361 | 0.5870 | 0.4562 |
| C | 0.0639 | 0.4130 | 0.5438 |
| C | 0.0639 | 0.9130 | 0.4562 |
| C | 0.9361 | 0.0870 | 0.5438 |
| C | 0.5639 | 0.0870 | 0.5438 |
| C | 0.4361 | 0.9130 | 0.4562 |
| F | 0.9994 | 0.9936 | 0.4002 |
| F | 0.0006 | 0.0064 | 0.5998 |
| F | 0.4994 | 0.0064 | 0.5998 |
| F | 0.5006 | 0.9936 | 0.4002 |
| F | 0.5006 | 0.4936 | 0.5998 |
| F | 0.4994 | 0.5064 | 0.4002 |
| F | 0.0006 | 0.5064 | 0.4002 |
| F | 0.9994 | 0.4936 | 0.5998 |
| Tx | 8.2242 | 0.0000 | 0.0000 |
| Ty | 0.0000 | 8.5843 | 0.0000 |

| | | | |
|---|---|---|---|
| Tz | 0.0000 | 0.0000 | 81.2582 |
| 4 layers | | | |
| Atom | X | Y | Z |
| C | 0.9322 | 0.4066 | 0.4042 |
| C | 0.0678 | 0.5934 | 0.5958 |
| C | 0.4322 | 0.5934 | 0.5958 |
| C | 0.5678 | 0.4066 | 0.4042 |
| C | 0.5678 | 0.9066 | 0.5958 |
| C | 0.4322 | 0.0934 | 0.4042 |
| C | 0.0678 | 0.0934 | 0.4042 |
| C | 0.9322 | 0.9066 | 0.5958 |
| C | 0.9379 | 0.4169 | 0.4582 |
| C | 0.0621 | 0.5831 | 0.5418 |
| C | 0.4379 | 0.5831 | 0.5418 |
| C | 0.5621 | 0.4169 | 0.4582 |
| C | 0.5621 | 0.9169 | 0.5418 |
| C | 0.4379 | 0.0831 | 0.4582 |
| C | 0.0621 | 0.0831 | 0.4582 |
| C | 0.9379 | 0.9169 | 0.5418 |
| C | 0.9377 | 0.4166 | 0.5139 |
| C | 0.0623 | 0.5834 | 0.4861 |
| C | 0.4377 | 0.5834 | 0.4861 |
| C | 0.5623 | 0.4166 | 0.5139 |
| C | 0.5623 | 0.9166 | 0.4861 |
| C | 0.4377 | 0.0834 | 0.5139 |
| C | 0.0623 | 0.0834 | 0.5139 |
| C | 0.9377 | 0.9166 | 0.4861 |
| C | 0.9359 | 0.4130 | 0.5697 |
| C | 0.0641 | 0.5870 | 0.4303 |
| C | 0.4359 | 0.5870 | 0.4303 |
| C | 0.5641 | 0.4130 | 0.5697 |
| C | 0.5641 | 0.9130 | 0.4303 |
| C | 0.4359 | 0.0870 | 0.5697 |
| C | 0.0641 | 0.0870 | 0.5697 |
| C | 0.9359 | 0.9130 | 0.4303 |
| F | 0.0002 | 0.4931 | 0.6233 |
| F | 0.9998 | 0.5069 | 0.3767 |
| F | 0.5002 | 0.5069 | 0.3767 |
| F | 0.4998 | 0.4931 | 0.6233 |
| F | 0.4998 | 0.9931 | 0.3767 |
| F | 0.5002 | 0.0069 | 0.6233 |
| F | 0.9998 | 0.0069 | 0.6233 |
| F | 0.0002 | 0.9931 | 0.3767 |
| Tx | 8.1496 | 0.0000 | 0.0000 |
| Ty | 0.0000 | 8.4985 | 0.0000 |
| Tz | 0.0000 | 0.0000 | 85.0377 |
| 5 layers | | | |
| Atom | X | Y | Z |
| C | 0.4323 | 0.4070 | 0.3815 |
| C | 0.5677 | 0.5930 | 0.6185 |

| | | | |
|---|---|---|---|
| C | 0.9323 | 0.5930 | 0.6185 |
| C | 0.0677 | 0.4070 | 0.3815 |
| C | 0.0677 | 0.9070 | 0.6185 |
| C | 0.9323 | 0.0930 | 0.3815 |
| C | 0.5677 | 0.0930 | 0.3815 |
| C | 0.4323 | 0.9070 | 0.6185 |
| C | 0.4378 | 0.4168 | 0.4333 |
| C | 0.5622 | 0.5832 | 0.5667 |
| C | 0.9378 | 0.5832 | 0.5667 |
| C | 0.0622 | 0.4168 | 0.4333 |
| C | 0.0622 | 0.9168 | 0.5667 |
| C | 0.9378 | 0.0832 | 0.4333 |
| C | 0.5622 | 0.0832 | 0.4333 |
| C | 0.4378 | 0.9168 | 0.5667 |
| C | 0.4376 | 0.4166 | 0.4867 |
| C | 0.5624 | 0.5834 | 0.5133 |
| C | 0.9376 | 0.5834 | 0.5133 |
| C | 0.0624 | 0.4166 | 0.4867 |
| C | 0.0624 | 0.9166 | 0.5133 |
| C | 0.9376 | 0.0834 | 0.4867 |
| C | 0.5624 | 0.0834 | 0.4867 |
| C | 0.4376 | 0.9166 | 0.5133 |
| C | 0.4376 | 0.4166 | 0.5400 |
| C | 0.5624 | 0.5834 | 0.4600 |
| C | 0.9376 | 0.5834 | 0.4600 |
| C | 0.0624 | 0.4166 | 0.5400 |
| C | 0.0624 | 0.9166 | 0.4600 |
| C | 0.9376 | 0.0834 | 0.5400 |
| C | 0.5624 | 0.0834 | 0.5400 |
| C | 0.4376 | 0.9166 | 0.4600 |
| C | 0.4357 | 0.4130 | 0.5934 |
| C | 0.5643 | 0.5870 | 0.4066 |
| C | 0.9357 | 0.5870 | 0.4066 |
| C | 0.0643 | 0.4130 | 0.5934 |
| C | 0.0643 | 0.9130 | 0.4066 |
| C | 0.9357 | 0.0870 | 0.5934 |
| C | 0.5643 | 0.0870 | 0.5934 |
| C | 0.4357 | 0.9130 | 0.4066 |
| F | 0.5001 | 0.5072 | 0.3552 |
| F | 0.4999 | 0.4928 | 0.6448 |
| F | 0.0001 | 0.4928 | 0.6448 |
| F | 0.9999 | 0.5072 | 0.3552 |
| F | 0.9999 | 0.0072 | 0.6448 |
| F | 0.0001 | 0.9928 | 0.3552 |
| F | 0.4999 | 0.9928 | 0.3552 |
| F | 0.5001 | 0.0072 | 0.6448 |
| Tx | 8.1030 | 0.0000 | 0.0000 |
| Ty | 0.0000 | 8.4458 | 0.0000 |
| Tz | 0.0000 | 0.0000 | 88.8171 |

6 layers

| Atom | X | Y | Z | Bader's Charges |
|---|---|---|---|---|
| C | 0.4324 | 0.9072 | 0.3635 | 0.801 |
| C | 0.5676 | 0.0928 | 0.6365 | 0.801 |
| C | 0.9324 | 0.0928 | 0.6365 | 0.801 |
| C | 0.0676 | 0.9072 | 0.3635 | 0.801 |
| C | 0.0676 | 0.4072 | 0.6365 | 0.801 |
| C | 0.9324 | 0.5928 | 0.3635 | 0.801 |
| C | 0.5676 | 0.5928 | 0.3635 | 0.801 |
| C | 0.4324 | 0.4072 | 0.6365 | 0.801 |
| C | 0.4377 | 0.9168 | 0.4122 | -0.007 |
| C | 0.5623 | 0.0832 | 0.5878 | -0.007 |
| C | 0.9377 | 0.0832 | 0.5878 | -0.007 |
| C | 0.0623 | 0.9168 | 0.4122 | -0.007 |
| C | 0.0623 | 0.4168 | 0.5878 | -0.007 |
| C | 0.9377 | 0.5832 | 0.4122 | -0.007 |
| C | 0.5623 | 0.5832 | 0.4122 | -0.007 |
| C | 0.4377 | 0.4168 | 0.5878 | -0.007 |
| C | 0.4375 | 0.9165 | 0.4624 | 0.000 |
| C | 0.5625 | 0.0835 | 0.5376 | 0.000 |
| C | 0.9375 | 0.0835 | 0.5376 | 0.000 |
| C | 0.0625 | 0.9165 | 0.4624 | 0.000 |
| C | 0.0625 | 0.4165 | 0.5376 | 0.000 |
| C | 0.9375 | 0.5835 | 0.4624 | 0.000 |
| C | 0.5625 | 0.5835 | 0.4624 | 0.000 |
| C | 0.4375 | 0.4165 | 0.5376 | 0.000 |
| C | 0.4375 | 0.9166 | 0.5125 | 0.000 |
| C | 0.5625 | 0.0834 | 0.4875 | 0.000 |
| C | 0.9375 | 0.0834 | 0.4875 | 0.000 |
| C | 0.0625 | 0.9166 | 0.5125 | 0.000 |
| C | 0.0625 | 0.4166 | 0.4875 | 0.000 |
| C | 0.9375 | 0.5834 | 0.5125 | 0.000 |
| C | 0.5625 | 0.5834 | 0.5125 | 0.000 |
| C | 0.4375 | 0.4166 | 0.4875 | 0.000 |
| C | 0.4375 | 0.9166 | 0.5627 | 0.001 |
| C | 0.5625 | 0.0834 | 0.4373 | 0.001 |
| C | 0.9375 | 0.0834 | 0.4373 | 0.001 |
| C | 0.0625 | 0.9166 | 0.5627 | 0.001 |
| C | 0.0625 | 0.4166 | 0.4373 | 0.001 |
| C | 0.9375 | 0.5834 | 0.5627 | 0.001 |
| C | 0.5625 | 0.5834 | 0.5627 | 0.001 |
| C | 0.4375 | 0.4166 | 0.4373 | 0.001 |
| C | 0.4357 | 0.9131 | 0.6129 | -0.002 |
| C | 0.5643 | 0.0869 | 0.3871 | -0.002 |
| C | 0.9357 | 0.0869 | 0.3871 | -0.002 |
| C | 0.0643 | 0.9131 | 0.6129 | -0.002 |
| C | 0.0643 | 0.4131 | 0.3871 | -0.002 |
| C | 0.9357 | 0.5869 | 0.6129 | -0.002 |
| C | 0.5643 | 0.5869 | 0.6129 | -0.002 |
| C | 0.4357 | 0.4131 | 0.3871 | -0.002 |
| F | 0.4998 | 0.9927 | 0.6613 | -0.793 |

| | | | | |
|---|---|---|---|---|
| F | 0.5002 | 0.0073 | 0.3387 | -0.793 |
| F | 0.9998 | 0.0073 | 0.3387 | -0.793 |
| F | 0.0002 | 0.9927 | 0.6613 | -0.793 |
| F | 0.0002 | 0.4927 | 0.3387 | -0.793 |
| F | 0.9998 | 0.5073 | 0.6613 | -0.793 |
| F | 0.5002 | 0.5073 | 0.6613 | -0.793 |
| F | 0.4998 | 0.4927 | 0.3387 | -0.793 |
| Tx | 8.0712 | 0.0000 | 0.0000 | |
| Ty | 0.0000 | 8.4103 | 0.0000 | |
| Tz | 0.0000 | 0.0000 | 94.4863 | |

**Table S10.** Information about H-($\bar{2}110$) films: atomic coordinates and lattice vectors.

| 1 layer | | | |
|---|---|---|---|
| Atom | X | Y | Z |
| C | 0.9319 | 0.9068 | 0.5153 |
| C | 0.0681 | 0.0932 | 0.4847 |
| C | 0.4319 | 0.0932 | 0.4847 |
| C | 0.5681 | 0.9068 | 0.5153 |
| C | 0.5681 | 0.4068 | 0.4847 |
| C | 0.4319 | 0.5932 | 0.5153 |
| C | 0.0681 | 0.5932 | 0.5153 |
| C | 0.9319 | 0.4068 | 0.4847 |
| H | 0.4956 | 0.5093 | 0.4595 |
| H | 0.5044 | 0.4907 | 0.5405 |
| H | 0.9956 | 0.4907 | 0.5405 |
| H | 0.0044 | 0.5093 | 0.4595 |
| H | 0.0044 | 0.0093 | 0.5405 |
| H | 0.9956 | 0.9907 | 0.4595 |
| H | 0.5044 | 0.9907 | 0.4595 |
| H | 0.4956 | 0.0093 | 0.5405 |
| Tx | 8.1504 | 0.0000 | 0.0000 |
| Ty | 0.0000 | 8.6049 | 0.0000 |
| Tz | 0.0000 | 0.0000 | 70.7627 |
| 2 layers | | | |
| Atom | X | Y | Z |
| C | 0.4374 | 0.9161 | 0.4842 |
| C | 0.5626 | 0.0839 | 0.5158 |
| C | 0.9374 | 0.0839 | 0.5158 |
| C | 0.0626 | 0.9161 | 0.4842 |
| C | 0.0626 | 0.4161 | 0.5158 |
| C | 0.9374 | 0.5839 | 0.4842 |
| C | 0.5626 | 0.5839 | 0.4842 |
| C | 0.4374 | 0.4161 | 0.5158 |
| C | 0.4330 | 0.9112 | 0.5463 |
| C | 0.5670 | 0.0888 | 0.4537 |
| C | 0.9330 | 0.0888 | 0.4537 |
| C | 0.0670 | 0.9112 | 0.5463 |
| C | 0.0670 | 0.4112 | 0.4537 |

| | | | |
|---|---|---|---|
| C | 0.9330 | 0.5888 | 0.5463 |
| C | 0.5670 | 0.5888 | 0.5463 |
| C | 0.4330 | 0.4112 | 0.4537 |
| H | 0.9880 | 0.5237 | 0.4307 |
| H | 0.0120 | 0.4763 | 0.5693 |
| H | 0.4880 | 0.4763 | 0.5693 |
| H | 0.5120 | 0.5237 | 0.4307 |
| H | 0.5120 | 0.0237 | 0.5693 |
| H | 0.4880 | 0.9763 | 0.4307 |
| H | 0.0120 | 0.9763 | 0.4307 |
| H | 0.9880 | 0.0237 | 0.5693 |
| Tx | 8.0076 | 0.0000 | 0.0000 |
| Ty | 0.0000 | 8.3341 | 0.0000 |
| Tz | 0.0000 | 0.0000 | 75.5890 |
| 3 layers | | | |
| Atom | X | Y | Z |
| C | 0.4332 | 0.4118 | 0.4277 |
| C | 0.5668 | 0.5882 | 0.5723 |
| C | 0.9332 | 0.5882 | 0.5723 |
| C | 0.0668 | 0.4118 | 0.4277 |
| C | 0.0668 | 0.9118 | 0.5723 |
| C | 0.9332 | 0.0882 | 0.4277 |
| C | 0.5668 | 0.0882 | 0.4277 |
| C | 0.4332 | 0.9118 | 0.5723 |
| C | 0.4375 | 0.4169 | 0.4854 |
| C | 0.5625 | 0.5831 | 0.5146 |
| C | 0.9375 | 0.5831 | 0.5146 |
| C | 0.0625 | 0.4169 | 0.4854 |
| C | 0.0625 | 0.9169 | 0.5146 |
| C | 0.9375 | 0.0831 | 0.4854 |
| C | 0.5625 | 0.0831 | 0.4854 |
| C | 0.4375 | 0.9169 | 0.5146 |
| C | 0.4372 | 0.4159 | 0.5439 |
| C | 0.5628 | 0.5841 | 0.4561 |
| C | 0.9372 | 0.5841 | 0.4561 |
| C | 0.0628 | 0.4159 | 0.5439 |
| C | 0.0628 | 0.9159 | 0.4561 |
| C | 0.9372 | 0.0841 | 0.5439 |
| C | 0.5628 | 0.0841 | 0.5439 |
| C | 0.4372 | 0.9159 | 0.4561 |
| H | 0.0131 | 0.9748 | 0.4064 |
| H | 0.9869 | 0.0252 | 0.5936 |
| H | 0.5131 | 0.0252 | 0.5936 |
| H | 0.4869 | 0.9748 | 0.4064 |
| H | 0.4869 | 0.4748 | 0.5936 |
| H | 0.5131 | 0.5252 | 0.4064 |
| H | 0.9869 | 0.5252 | 0.4064 |
| H | 0.0131 | 0.4748 | 0.5936 |
| Tx | 7.9711 | 0.0000 | 0.0000 |
| Ty | 0.0000 | 8.2924 | 0.0000 |

| | | | |
|---|---|---|---|
| Tz | 0.0000 | 0.0000 | 81.2582 |
| 4 layers | | | |
| Atom | X | Y | Z |
| C | 0.9334 | 0.4120 | 0.4029 |
| C | 0.0666 | 0.5880 | 0.5971 |
| C | 0.4334 | 0.5880 | 0.5971 |
| C | 0.5666 | 0.4120 | 0.4029 |
| C | 0.5666 | 0.9120 | 0.5971 |
| C | 0.4334 | 0.0880 | 0.4029 |
| C | 0.0666 | 0.0880 | 0.4029 |
| C | 0.9334 | 0.9120 | 0.5971 |
| C | 0.9375 | 0.4169 | 0.4581 |
| C | 0.0625 | 0.5831 | 0.5419 |
| C | 0.4375 | 0.5831 | 0.5419 |
| C | 0.5625 | 0.4169 | 0.4581 |
| C | 0.5625 | 0.9169 | 0.5419 |
| C | 0.4375 | 0.0831 | 0.4581 |
| C | 0.0625 | 0.0831 | 0.4581 |
| C | 0.9375 | 0.9169 | 0.5419 |
| C | 0.9373 | 0.4166 | 0.5140 |
| C | 0.0627 | 0.5834 | 0.4861 |
| C | 0.4373 | 0.5834 | 0.4861 |
| C | 0.5627 | 0.4166 | 0.5140 |
| C | 0.5627 | 0.9166 | 0.4861 |
| C | 0.4373 | 0.0834 | 0.5140 |
| C | 0.0627 | 0.0834 | 0.5140 |
| C | 0.9373 | 0.9166 | 0.4861 |
| C | 0.9371 | 0.4159 | 0.5698 |
| C | 0.0629 | 0.5841 | 0.4302 |
| C | 0.4371 | 0.5841 | 0.4302 |
| C | 0.5629 | 0.4159 | 0.5698 |
| C | 0.5629 | 0.9159 | 0.4302 |
| C | 0.4371 | 0.0841 | 0.5698 |
| C | 0.0629 | 0.0841 | 0.5698 |
| C | 0.9371 | 0.9159 | 0.4302 |
| H | 0.9863 | 0.4741 | 0.6174 |
| H | 0.0137 | 0.5259 | 0.3826 |
| H | 0.4863 | 0.5259 | 0.3826 |
| H | 0.5137 | 0.4741 | 0.6174 |
| H | 0.5137 | 0.9741 | 0.3826 |
| H | 0.4863 | 0.0259 | 0.6174 |
| H | 0.0137 | 0.0259 | 0.6174 |
| H | 0.9863 | 0.9741 | 0.3826 |
| Tx | 7.9532 | 0.0000 | 0.0000 |
| Ty | 0.0000 | 8.2733 | 0.0000 |
| Tz | 0.0000 | 0.0000 | 85.0377 |
| 5 layers | | | |
| Atom | X | Y | Z |
| C | 0.4334 | 0.4121 | 0.3803 |
| C | 0.5666 | 0.5879 | 0.6197 |

| | | | |
|---|---|---|---|
| C | 0.9334 | 0.5879 | 0.6197 |
| C | 0.0666 | 0.4121 | 0.3803 |
| C | 0.0666 | 0.9121 | 0.6197 |
| C | 0.9334 | 0.0879 | 0.3803 |
| C | 0.5666 | 0.0879 | 0.3803 |
| C | 0.4334 | 0.9121 | 0.6197 |
| C | 0.4375 | 0.4169 | 0.4332 |
| C | 0.5625 | 0.5831 | 0.5668 |
| C | 0.9375 | 0.5831 | 0.5668 |
| C | 0.0625 | 0.4169 | 0.4332 |
| C | 0.0625 | 0.9169 | 0.5668 |
| C | 0.9375 | 0.0831 | 0.4332 |
| C | 0.5625 | 0.0831 | 0.4332 |
| C | 0.4375 | 0.9169 | 0.5668 |
| C | 0.4373 | 0.4166 | 0.4866 |
| C | 0.5627 | 0.5834 | 0.5134 |
| C | 0.9373 | 0.5834 | 0.5134 |
| C | 0.0627 | 0.4166 | 0.4866 |
| C | 0.0627 | 0.9166 | 0.5134 |
| C | 0.9373 | 0.0834 | 0.4866 |
| C | 0.5627 | 0.0834 | 0.4866 |
| C | 0.4373 | 0.9166 | 0.5134 |
| C | 0.4373 | 0.4166 | 0.5401 |
| C | 0.5627 | 0.5834 | 0.4599 |
| C | 0.9373 | 0.5834 | 0.4599 |
| C | 0.0627 | 0.4166 | 0.5401 |
| C | 0.0627 | 0.9166 | 0.4599 |
| C | 0.9373 | 0.0834 | 0.5401 |
| C | 0.5627 | 0.0834 | 0.5401 |
| C | 0.4373 | 0.9166 | 0.4599 |
| C | 0.4371 | 0.4160 | 0.5936 |
| C | 0.5629 | 0.5840 | 0.4064 |
| C | 0.9371 | 0.5840 | 0.4064 |
| C | 0.0629 | 0.4160 | 0.5936 |
| C | 0.0629 | 0.9160 | 0.4064 |
| C | 0.9371 | 0.0840 | 0.5936 |
| C | 0.5629 | 0.0840 | 0.5936 |
| C | 0.4371 | 0.9160 | 0.4064 |
| H | 0.5140 | 0.5263 | 0.3609 |
| H | 0.4860 | 0.4737 | 0.6391 |
| H | 0.0140 | 0.4737 | 0.6391 |
| H | 0.9860 | 0.5263 | 0.3609 |
| H | 0.9860 | 0.0263 | 0.6391 |
| H | 0.0140 | 0.9737 | 0.3609 |
| H | 0.4860 | 0.9737 | 0.3609 |
| H | 0.5140 | 0.0263 | 0.6391 |
| Tx | 7.9424 | 0.0000 | 0.0000 |
| Ty | 0.0000 | 8.2625 | 0.0000 |
| Tz | 0.0000 | 0.0000 | 88.8171 |

| 6 layers |
|---|

| Atom | X | Y | Z | Bader's Charges |
|---|---|---|---|---|
| C | 0.4334 | 0.9122 | 0.3624 | -0.015 |
| C | 0.5666 | 0.0878 | 0.6376 | -0.015 |
| C | 0.9334 | 0.0878 | 0.6376 | -0.015 |
| C | 0.0666 | 0.9122 | 0.3624 | -0.015 |
| C | 0.0666 | 0.4122 | 0.6376 | -0.015 |
| C | 0.9334 | 0.5878 | 0.3624 | -0.015 |
| C | 0.5666 | 0.5878 | 0.3624 | -0.015 |
| C | 0.4334 | 0.4122 | 0.6376 | -0.015 |
| C | 0.4374 | 0.9169 | 0.4121 | -0.003 |
| C | 0.5626 | 0.0831 | 0.5879 | -0.003 |
| C | 0.9374 | 0.0831 | 0.5879 | -0.003 |
| C | 0.0626 | 0.9169 | 0.4121 | -0.003 |
| C | 0.0626 | 0.4169 | 0.5879 | -0.003 |
| C | 0.9374 | 0.5831 | 0.4121 | -0.003 |
| C | 0.5626 | 0.5831 | 0.4121 | -0.003 |
| C | 0.4374 | 0.4169 | 0.5879 | -0.003 |
| C | 0.4373 | 0.9166 | 0.4623 | 0.000 |
| C | 0.5627 | 0.0834 | 0.5377 | 0.000 |
| C | 0.9373 | 0.0834 | 0.5377 | 0.000 |
| C | 0.0627 | 0.9166 | 0.4623 | 0.000 |
| C | 0.0627 | 0.4166 | 0.5377 | 0.000 |
| C | 0.9373 | 0.5834 | 0.4623 | 0.000 |
| C | 0.5627 | 0.5834 | 0.4623 | 0.000 |
| C | 0.4373 | 0.4166 | 0.5377 | 0.000 |
| C | 0.4373 | 0.9166 | 0.5126 | 0.000 |
| C | 0.5627 | 0.0834 | 0.4874 | 0.000 |
| C | 0.9373 | 0.0834 | 0.4874 | 0.000 |
| C | 0.0627 | 0.9166 | 0.5126 | 0.000 |
| C | 0.0627 | 0.4166 | 0.4874 | 0.000 |
| C | 0.9373 | 0.5834 | 0.5126 | 0.000 |
| C | 0.5627 | 0.5834 | 0.5126 | 0.000 |
| C | 0.4373 | 0.4166 | 0.4874 | 0.000 |
| C | 0.4373 | 0.9166 | 0.5628 | 0.000 |
| C | 0.5627 | 0.0834 | 0.4372 | 0.000 |
| C | 0.9373 | 0.0834 | 0.4372 | 0.000 |
| C | 0.0627 | 0.9166 | 0.5628 | 0.000 |
| C | 0.0627 | 0.4166 | 0.4372 | 0.000 |
| C | 0.9373 | 0.5834 | 0.5628 | 0.000 |
| C | 0.5627 | 0.5834 | 0.5628 | 0.000 |
| C | 0.4373 | 0.4166 | 0.4372 | 0.000 |
| C | 0.4371 | 0.9160 | 0.6131 | 0.003 |
| C | 0.5629 | 0.0840 | 0.3869 | 0.003 |
| C | 0.9371 | 0.0840 | 0.3869 | 0.003 |
| C | 0.0629 | 0.9160 | 0.6131 | 0.003 |
| C | 0.0629 | 0.4160 | 0.3869 | 0.003 |
| C | 0.9371 | 0.5840 | 0.6131 | 0.003 |
| C | 0.5629 | 0.5840 | 0.6131 | 0.003 |
| C | 0.4371 | 0.4160 | 0.3869 | 0.003 |
| H | 0.4858 | 0.9735 | 0.6559 | 0.015 |

| | | | | |
|---|---|---|---|---|
| H | 0.5142 | 0.0265 | 0.3441 | 0.015 |
| H | 0.9858 | 0.0265 | 0.3441 | 0.015 |
| H | 0.0142 | 0.9735 | 0.6559 | 0.015 |
| H | 0.0142 | 0.4735 | 0.3441 | 0.015 |
| H | 0.9858 | 0.5265 | 0.6559 | 0.015 |
| H | 0.5142 | 0.5265 | 0.6559 | 0.015 |
| H | 0.4858 | 0.4735 | 0.3441 | 0.015 |
| Tx | 7.9354 | 0.0000 | 0.0000 | |
| Ty | 0.0000 | 8.2555 | 0.0000 | |
| Tz | 0.0000 | 0.0000 | 94.4863 | |

**Table S11.** Structural and electronic properties of studied hydrogenated diamanes

| C-H | d, Bohr | ΔX | $\mu_{TO}$ | Fundamental gap (TB09), eV | Fundamental gap (GW@TB09), eV | Direct gap (TB09), eV | Direct gap (GW@TB09), eV |
|---|---|---|---|---|---|---|---|
| 1H-(0001) | 2.0946 | 0.11 | 0.2304 | 2.92 | 9.33 | 2.92 | 9.33 |
| 2H-(0001) | 2.0912 | 0.11 | 0.2300 | 2.56 | 8.46 | 2.56 | 8.46 |
| 3H-(0001) | 2.0913 | 0.11 | 0.2300 | 2.47 | 8.17 | 2.47 | 8.17 |
| 4H-(0001) | 2.0913 | 0.11 | 0.2300 | 2.52 | 7.88 | 2.52 | 7.88 |
| 5H-(0001) | 2.0913 | 0.11 | 0.2300 | 2.58 | 7.64 | 2.58 | 7.64 |
| 6H-(0001) | 2.0913 | 0.11 | 0.2300 | 2.72 | 7.29 | 2.72 | 7.29 |
| | | | | | | | |
| C-H | d, Bohr | ΔX | $\mu_{TO}$ | Fundamental gap (TB09), eV | Fundamental gap (GW@TB09), eV | Direct gap (TB09), eV | Direct gap (GW@TB09), eV |
| 1H-(110) | 2.0848 | 0.11 | 0.2293 | 2.72 | 8.83 | 2.72 | 8.84 |
| 2H-(110) | 2.0785 | 0.11 | 0.2286 | 2.37 | 8.24 | 2.37 | 8.24 |
| 3H-(110) | 2.0778 | 0.11 | 0.2286 | 1.96 | 7.77 | 1.96 | 7.77 |
| 4H-(110) | 2.0774 | 0.11 | 0.2285 | 2.01 | 7.62 | 2.01 | 7.62 |
| 5H-(110) | 2.0767 | 0.11 | 0.2284 | 2.91 | 7.45 | 2.91 | 7.45 |
| 6H-(110) | 2.0765 | 0.11 | 0.2284 | 2.98 | 7.41 | 2.98 | 7.41 |
| | | | | | | | |
| C-H | d, Bohr | ΔX | $\mu_{TO}$ | Fundamental gap (TB09), eV | Fundamental gap (GW@TB09), eV | Direct gap (TB09), eV | Direct gap (GW@TB09), eV |
| 1H-(111) | 2.0945 | 0.11 | 0.2304 | 2.92 | 9.33 | 2.92 | 9.33 |
| 2H-(111) | 2.0932 | 0.11 | 0.2303 | 2.77 | 8.63 | 2.77 | 8.63 |
| 3H-(111) | 2.0937 | 0.11 | 0.2303 | 2.75 | 8.41 | 2.75 | 8.41 |
| 4H-(111) | 2.0939 | 0.11 | 0.2303 | 2.84 | 8.16 | 2.84 | 8.16 |
| 5H-(111) | 2.0940 | 0.11 | 0.2303 | 2.91 | 7.94 | 2.91 | 7.94 |
| 6H-(111) | 2.0940 | 0.11 | 0.2303 | 2.98 | 7.74 | 2.98 | 7.74 |
| | | | | | | | |
| C-H | d, Bohr | ΔX | $\mu_{TO}$ | Fundamental gap (TB09), eV | Fundamental gap (GW@TB09), eV | Direct gap (TB09), eV | Direct gap (GW@TB09), eV |
| 1H-$(10\bar{1}0)$ | 2.0839 | 0.11 | 0.2292 | 2.82 | 9.36 | 2.82 | 9.36 |
| 2H-$(10\bar{1}0)$ | 2.0796 | 0.11 | 0.2288 | 2.37 | 8.45 | 2.37 | 8.45 |
| 3H-$(10\bar{1}0)$ | 2.0801 | 0.11 | 0.2288 | 2.33 | 8.36 | 2.33 | 8.36 |
| 4H-$(10\bar{1}0)$ | 2.0792 | 0.11 | 0.2287 | 2.21 | 7.77 | 2.21 | 7.77 |

| | | | | | | | |
|---|---|---|---|---|---|---|---|
| 5H-$(10\bar{1}0)$ | 2.0789 | 0.11 | 0.2287 | 2.16 | 7.62 | 2.16 | 7.62 |
| 6H-$(10\bar{1}0)$ | 2.0788 | 0.11 | 0.2287 | 2.21 | 7.39 | 2.21 | 7.39 |
| | | | | | | | |
| C-H | d, Bohr | ΔX | $\mu_{TO}$ | Fundamental gap (TB09), eV | Fundamental gap (GW@TB09), eV | Direct gap (TB09), eV | Direct gap (GW@TB09), eV |
| 1H-$(\bar{2}110)$ | 2.0783 | 0.11 | 0.2286 | 2.63 | 6.37 | 2.63 | 6.37 |
| 2H-$(\bar{2}110)$ | 2.0726 | 0.11 | 0.2280 | 2.15 | 5.78 | 2.15 | 5.78 |
| 3H-$(\bar{2}110)$ | 2.0718 | 0.11 | 0.2279 | 2.06 | 5.32 | 2.06 | 5.32 |
| 4H-$(\bar{2}110)$ | 2.0714 | 0.11 | 0.2279 | 2.07 | 5.13 | 2.07 | 5.13 |
| 5H-$(\bar{2}110)$ | 2.0712 | 0.11 | 0.2278 | 2.12 | 5.04 | 2.12 | 5.04 |
| 6H-$(\bar{2}110)$ | 2.0710 | 0.11 | 0.2278 | 2.14 | 4.96 | 2.14 | 4.97 |

**Table S12.** Structural and electronic properties of studied fluorinated diamanes

| C-F | d, Bohr | ΔX | $\mu_{TO}$ | Fundamental gap (TB09), eV | Fundamental gap (GW@TB09), eV | Direct gap (TB09), eV | Direct gap (GW@TB09), eV |
|---|---|---|---|---|---|---|---|
| 1F-(0001) | 2.5930 | 0.96 | 2.4893 | 3.16 | 9.63 | 3.16 | 9.63 |
| 2F-(0001) | 2.5838 | 0.96 | 2.4804 | 4.1 | 10.39 | 4.1 | 10.01 |
| 3F-(0001) | 2.5840 | 0.96 | 2.4807 | 4.28 | 9.95 | 4.37 | 10.12 |
| 4F-(0001) | 2.5848 | 0.96 | 2.4814 | 4.2 | 9.59 | 4.57 | 10.02 |
| 5F-(0001) | 2.5841 | 0.96 | 2.4808 | 4.17 | 9.33 | 4.7 | 9.89 |
| 6F-(0001) | 2.5839 | 0.96 | 2.4806 | 4.21 | 8.96 | 4.85 | 9.59 |
| | | | | | | | |
| C-F | d, Bohr | ΔX | $\mu_{TO}$ | Fundamental gap (TB09), eV | Fundamental gap (GW@TB09), eV | Direct gap (TB09), eV | Direct gap (GW@TB09), eV |
| 1F-(110) | 2.5937 | 0.96 | 2.4900 | 3.53 | 9.41 | 3.53 | 9.41 |
| 2F-(110) | 2.5609 | 0.96 | 2.4585 | 4.52 | 9.91 | 4.52 | 9.91 |
| 3F-(110) | 2.5619 | 0.96 | 2.4594 | 4.31 | 9.75 | 4.31 | 9.75 |
| 4F-(110) | 2.5595 | 0.96 | 2.4571 | 4.4 | 9.69 | 4.5 | 9.72 |
| 5F-(110) | 2.5582 | 0.96 | 2.4558 | 4.19 | 9.27 | 4.53 | 9.68 |
| 6F-(110) | 2.5383 | 0.96 | 2.4368 | 4.15 | 9.02 | 4.99 | 9.54 |
| | | | | | | | |
| C-F | d, Bohr | ΔX | $\mu_{TO}$ | Fundamental gap (TB09), eV | Fundamental gap (GW@TB09), eV | Direct gap (TB09), eV | Direct gap (GW@TB09), eV |
| 1F-(111) | 2.5931 | 0.96 | 2.4894 | 3.17 | 9.62 | 3.17 | 9.62 |
| 2F-(111) | 2.5863 | 0.96 | 2.4829 | 4.14 | 10.46 | 4.14 | 10.02 |
| 3F-(111) | 2.5873 | 0.96 | 2.4838 | 4.32 | 10.03 | 4.48 | 10.20 |
| 4F-(111) | 2.5875 | 0.96 | 2.4840 | 4.25 | 9.679 | 4.72 | 10.16 |
| 5F-(111) | 2.5709 | 0.96 | 2.4680 | 4.27 | 9.47 | 4.77 | 9.97 |
| 6F-(111) | 2.5877 | 0.96 | 2.4842 | 4.15 | 9.09 | 4.99 | 9.93 |
| | | | | | | | |
| C-F | d, Bohr | ΔX | $\mu_{TO}$ | Fundamental gap (TB09), eV | Fundamental gap (GW@TB09), eV | Direct gap (TB09), eV | Direct gap (GW@TB09), eV |
| 1F-$(10\bar{1}0)$ | 2.5827 | 0.96 | 2.4794 | 3.13 | 9.49 | 3.13 | 9.49 |
| 2F-$(10\bar{1}0)$ | 2.5674 | 0.96 | 2.4647 | 4.03 | 9.89 | 4.03 | 9.89 |
| 3F-$(10\bar{1}0)$ | 2.5676 | 0.96 | 2.4649 | 4.00 | 9.93 | 4.0 | 9.93 |
| 4F-$(10\bar{1}0)$ | 2.5663 | 0.96 | 2.4637 | 3.69 | 9.22 | 4.05 | 9.55 |

| | | | | | | | |
|---|---|---|---|---|---|---|---|
| 5F-($10\bar{1}0$) | 2.5662 | 0.96 | 2.4635 | 3.43 | 8.91 | 3.99 | 9.46 |
| 6F-($10\bar{1}0$) | 2.5659 | 0.96 | 2.4633 | 3.31 | 8.54 | 4.03 | 9.25 |
| | | | | | | | |
| C-F | d, Bohr | ΔX | $\mu_{TO}$ | Fundamental gap (TB09), eV | Fundamental gap (GW@TB09), eV | Direct gap (TB09), eV | Direct gap (GW@TB09), eV |
| 1F-($\bar{2}110$) | 2.5820 | 0.96 | 2.4787 | 4.13 | 9.36 | 4.13 | 9.36 |
| 2F-($\bar{2}110$) | 2.5549 | 0.96 | 2.4527 | 4.84 | 9.36 | 4.84 | 9.36 |
| 3F-($\bar{2}110$) | 2.5519 | 0.96 | 2.4498 | 4.45 | 9.02 | 4.45 | 9.02 |
| 4F-($\bar{2}110$) | 2.5502 | 0.96 | 2.4482 | 4.07 | 7.89 | 4.07 | 7.89 |
| 5F-($\bar{2}110$) | 2.5491 | 0.96 | 2.4471 | 3.79 | 7.49 | 3.79 | 7.49 |
| 6F-($\bar{2}110$) | 2.5483 | 0.96 | 2.4464 | 3.29 | 7.24 | 3.29 | 7.24 |

**Table S13**. Calculated valence band maximum (VBM) energies, potentials at vacuum and work functions for considered diamanes.

| C-F | VBM energy (PBE), eV | VBM energy (GW@PBE), eV | VBM energy (GW@TB09), eV | Potential (PBE), eV | Work function (PBE), eV | Work function (GW@PBE), eV |
|---|---|---|---|---|---|---|
| 1F-(0001) | -6.150 | -9.317 | -10.532 | 1.560 | 7.710 | 10.877 |
| 2F-(0001) | -5.181 | -10.577 | -11.476 | 2.405 | 7.586 | 12.982 |
| 3F-(0001) | -4.529 | -10.279 | -11.031 | 3.092 | 7.621 | 13.371 |
| 4F-(0001) | -3.841 | -9.696 | -10.222 | 3.685 | 7.526 | 13.381 |
| 5F-(0001) | -3.207 | -9.136 | -9.469 | 4.180 | 7.387 | 13.316 |
| 6F-(0001) | -2.417 | -8.226 | -8.286 | 4.548 | 6.965 | 12.774 |
| C-F | VBM energy (PBE), eV | VBM energy (GW@PBE), eV | VBM energy (GW@TB09), eV | Potential (PBE), eV | Work function (PBE), eV | Work function (GW@PBE), eV |
| 1F-(110) | -6.231 | -8.876 | -10.034 | 1.600 | 7.831 | 10.476 |
| 2F-(110) | -5.651 | -8.303 | -9.363 | 2.210 | 7.861 | 10.513 |
| 3F-(110) | -5.274 | -8.000 | -8.948 | 2.660 | 7.934 | 10.660 |
| 4F-(110) | -4.858 | -7.646 | -8.494 | 3.070 | 7.928 | 10.716 |
| 5F-(110) | -4.494 | -7.308 | -8.080 | 3.440 | 7.934 | 10.748 |
| 6F-(110) | -4.160 | -7.070 | -7.748 | 3.780 | 7.940 | 10.850 |
| C-F | VBM energy (PBE), eV | VBM energy (GW@PBE), eV | VBM energy (GW@TB09), eV | Potential (PBE), eV | Work function (PBE), eV | Work function (GW@PBE), eV |
| 1F-(111) | -6.146 | -9.306 | -10.517 | 1.520 | 7.666 | 10.826 |
| 2F-(111) | -5.324 | -9.386 | -10.860 | 2.410 | 7.734 | 11.796 |
| 3F-(111) | -4.736 | -9.882 | -10.661 | 3.092 | 7.828 | 12.974 |
| 4F-(111) | -4.077 | -9.303 | -9.866 | 3.718 | 7.795 | 13.021 |
| 5F-(111) | -3.352 | -8.720 | -9.067 | 4.189 | 7.541 | 12.909 |
| 6F-(111) | -2.903 | -8.215 | -8.402 | 4.548 | 7.450 | 12.763 |
| C-F | VBM energy (PBE), eV | VBM energy (GW@PBE), eV | VBM energy (GW@TB09), eV | Potential (PBE), eV | Work function (PBE), eV | Work function (GW@PBE), eV |
| 1F-($10\bar{1}0$) | -6.120 | -12.088 | -13.189 | 1.550 | 7.670 | 13.638 |
| 2F-($10\bar{1}0$) | -5.162 | -8.255 | -9.265 | 2.432 | 7.594 | 10.687 |
| 3F-($10\bar{1}0$) | -4.461 | -7.285 | -9.099 | 3.178 | 7.639 | 10.463 |
| 4F-($10\bar{1}0$) | -3.720 | -6.960 | -7.603 | 3.865 | 7.585 | 10.825 |
| 5F-($10\bar{1}0$) | -3.230 | -6.623 | -7.166 | 4.340 | 7.570 | 10.963 |
| 6F-($10\bar{1}0$) | -2.633 | -6.023 | -6.382 | 4.880 | 7.513 | 10.903 |
| C-F | VBM energy (PBE), eV | VBM energy (GW@PBE), eV | VBM energy (GW@TB09), eV | Potential (PBE), eV | Work function (PBE), eV | Work function (GW@PBE), eV |

| | | | | | | |
|---|---|---|---|---|---|---|
| 1F-($\bar{2}110$) | -6.766 | -8.236 | -9.383 | 1.668 | 8.435 | 9.904 |
| 2F-($\bar{2}110$) | -5.615 | -6.851 | -5.465 | 2.712 | 8.327 | 9.563 |
| 3F-($\bar{2}110$) | -4.593 | -5.825 | -4.208 | 3.592 | 8.185 | 9.417 |
| 4F-($\bar{2}110$) | -3.596 | -4.777 | -2.896 | 4.426 | 8.021 | 9.203 |
| 5F-($\bar{2}110$) | -2.053 | -4.001 | -1.761 | 5.150 | 7.203 | 9.151 |
| 6F-($\bar{2}110$) | -0.444 | -3.207 | -0.973 | 5.777 | 6.221 | 8.984 |
| C-H | VBM energy (PBE), eV | VBM energy (GW@PBE), eV | VBM energy (GW@TB09), eV | Potential (PBE), eV | Work function (PBE), eV | Work function (GW@PBE), eV |
| 1H-(0001) | -3.876 | -6.855 | -8.653 | 0.800 | 4.676 | 7.655 |
| 2H-(0001) | -2.408 | -5.330 | -6.958 | 1.338 | 3.746 | 6.668 |
| 3H-(0001) | -1.552 | -4.610 | -6.065 | 1.852 | 3.404 | 6.462 |
| 4H-(0001) | -0.843 | -3.896 | -5.163 | 2.309 | 3.152 | 6.205 |
| 5H-(0001) | -0.254 | -3.307 | -4.380 | 2.718 | 2.972 | 6.025 |
| 6H-(0001) | 0.402 | -2.496 | -3.288 | 3.081 | 2.679 | 5.577 |
| C-H | VBM energy (PBE), eV | VBM energy (GW@PBE), eV | VBM energy (GW@TB09), eV | Potential (PBE), eV | Work function (PBE), eV | Work function (GW@PBE), eV |
| 1H-(110) | -3.692 | -6.353 | -8.091 | 0.920 | 4.612 | 7.273 |
| 2H-(110) | -2.542 | -5.243 | -6.884 | 1.242 | 3.784 | 6.485 |
| 3H-(110) | -1.838 | -4.579 | -6.123 | 1.544 | 3.381 | 6.123 |
| 4H-(110) | -1.335 | -4.136 | -5.468 | 1.775 | 3.109 | 5.911 |
| 5H-(110) | -0.944 | -3.759 | -5.094 | 1.963 | 2.907 | 5.722 |
| 6H-(110) | -0.621 | -3.545 | -4.778 | 2.100 | 2.721 | 5.645 |
| C-H | VBM energy (PBE), eV | VBM energy (GW@PBE), eV | VBM energy (GW@TB09), eV | Potential (PBE), eV | Work function (PBE), eV | Work function (GW@PBE), eV |
| 1H-(111) | -3.875 | -6.847 | -8.643 | 0.800 | 4.675 | 7.647 |
| 2H-(111) | -2.597 | -5.522 | -7.145 | 1.399 | 3.996 | 6.921 |
| 3H-(111) | -1.818 | -4.875 | -6.343 | 1.916 | 3.733 | 6.791 |
| 4H-(111) | -1.145 | -4.209 | -5.468 | 2.370 | 3.515 | 6.579 |
| 5H-(111) | -0.576 | -3.638 | -4.702 | 2.769 | 3.346 | 6.407 |
| 6H-(111) | -0.078 | -3.142 | -4.016 | 3.100 | 3.178 | 6.242 |
| C-H | VBM energy (PBE), eV | VBM energy (GW@PBE), eV | VBM energy (GW@TB09), eV | Potential (PBE), eV | Work function (PBE), eV | Work function (GW@PBE), eV |
| 1H-($10\bar{1}0$) | -3.711 | -6.770 | -8.561 | 0.800 | 4.511 | 7.570 |
| 2H-($10\bar{1}0$) | -2.170 | -5.224 | -6.821 | 1.396 | 3.566 | 6.620 |
| 3H-($10\bar{1}0$) | -1.327 | -4.488 | -6.461 | 1.904 | 3.231 | 6.392 |
| 4H-($10\bar{1}0$) | -0.554 | -3.748 | -4.944 | 2.398 | 2.952 | 6.146 |
| 5H-($10\bar{1}0$) | -0.071 | -3.419 | -4.478 | 2.822 | 2.893 | 6.241 |
| 6H-($10\bar{1}0$) | 0.442 | -2.904 | -3.771 | 3.200 | 2.758 | 6.104 |
| C-H | VBM energy (PBE), eV | VBM energy (GW@PBE), eV | VBM energy (GW@TB09), eV | Potential (PBE), eV | Work function (PBE), eV | Work function (GW@PBE), eV |
| 1H-($\bar{2}110$) | -3.701 | -5.545 | -5.181 | 0.890 | 4.591 | 6.435 |
| 2H-($\bar{2}110$) | -1.927 | -3.355 | -2.963 | 1.611 | 3.538 | 4.966 |
| 3H-($\bar{2}110$) | -0.897 | -2.082 | -1.430 | 2.251 | 3.149 | 4.333 |
| 4H-($\bar{2}110$) | -0.025 | -1.174 | -0.217 | 2.835 | 2.860 | 4.009 |
| 5H-($\bar{2}110$) | 0.682 | -0.454 | 0.777 | 3.369 | 2.687 | 3.823 |
| 6H-($\bar{2}110$) | 1.206 | 0.623 | 1.474 | 3.840 | 2.634 | 3.217 |

**Table S14**. Thickness of each slab, defined as distance between the H (or F) layers on the opposite sides of the considered diamane.

| C-F | Thickness, Å | C-H | Thickness, Å | C-F | Thickness, Å | C-H | Thickness, Å |
|---|---|---|---|---|---|---|---|
| 1F-(0001) | 3.236 | 1H-(0001) | 2.675 | 1F-(111) | 3.236 | 1H-(111) | 2.676 |
| 2F-(0001) | 5.322 | 2H-(0001) | 4.781 | 2F-(111) | 5.299 | 2H-(111) | 4.748 |
| 3F-(0001) | 7.413 | 3H-(0001) | 6.872 | 3F-(111) | 7.363 | 3H-(111) | 6.812 |
| 4F-(0001) | 9.504 | 4H-(0001) | 8.963 | 4F-(111) | 9.426 | 4H-(111) | 8.874 |
| 5F-(0001) | 11.592 | 5H-(0001) | 11.053 | 5F-(111) | 11.503 | 5H-(111) | 10.937 |
| 6F-(0001) | 13.685 | 6H-(0001) | 13.145 | 6F-(111) | 13.550 | 6H-(111) | 12.999 |
| C-F | Thickness, Å | C-H | Thickness, Å | C-F | Thickness, Å | C-H | Thickness, Å |
| 1F-(110) | 3.525 | 1H-(110) | 3.049 | 1F-$(10\bar{1}0)$ | 3.298 | 1H-$(10\bar{1}0)$ | 2.761 |
| 2F-(110) | 4.824 | 2H-(110) | 4.315 | 2F-$(10\bar{1}0)$ | 5.479 | 2H-$(10\bar{1}0)$ | 4.942 |
| 3F-(110) | 6.083 | 3H-(110) | 5.575 | 3F-$(10\bar{1}0)$ | 7.643 | 3H-$(10\bar{1}0)$ | 7.108 |
| 4F-(110) | 7.354 | 4H-(110) | 6.837 | 4F-$(10\bar{1}0)$ | 9.836 | 4H-$(10\bar{1}0)$ | 9.294 |
| 5F-(110) | 8.621 | 5H-(110) | 8.099 | 5F-$(10\bar{1}0)$ | 12.013 | 5H-$(10\bar{1}0)$ | 11.469 |
| 6F-(110) | 9.887 | 6H-(110) | 9.362 | 6F-$(10\bar{1}0)$ | 14.189 | 6H-$(10\bar{1}0)$ | 13.645 |
| C-F | Thickness, Å | C-H | Thickness, Å | | | | |
| 1F-$(\bar{2}110)$ | 3.515 | 1H-$(\bar{2}110)$ | 3.034 | | | | |
| 2F-$(\bar{2}110)$ | 6.057 | 2H-$(\bar{2}110)$ | 5.543 | | | | |
| 3F-$(\bar{2}110)$ | 8.579 | 3H-$(\bar{2}110)$ | 8.054 | | | | |
| 4F-$(\bar{2}110)$ | 11.097 | 4H-$(\bar{2}110)$ | 10.566 | | | | |
| 5F-$(\bar{2}110)$ | 13.612 | 5H-$(\bar{2}110)$ | 13.079 | | | | |
| 6F-$(\bar{2}110)$ | 16.127 | 6H-$(\bar{2}110)$ | 15.591 | | | | |

**Table S15.** Charge electron density coming from Bader's analysis at the VBM and levels below (VBM-N) projected to total number of carbon and hydrogen (fluorine) atoms for each surface.

| Hydrogenated | | | | | | | | | | |
|---|---|---|---|---|---|---|---|---|---|---|
| VBM-N | (0001) | | (111) | | (110) | | $(10\bar{1}0)$ | | $(\bar{2}110)$ | |
| | C | H | C | H | C | H | C | H | C | H |
| N=0 | 1.896 | 0.104 | 1.948 | 0.052 | 1.842 | 0.158 | 1.951 | 0.049 | 1.952 | 0.048 |
| N=1 | 1.917 | 0.083 | 1.833 | 0.167 | 1.860 | 0.140 | 1.922 | 0.078 | 1.952 | 0.048 |
| 2 | 1.810 | 0.190 | 1.942 | 0.058 | 1.845 | 0.155 | 1.931 | 0.069 | 1.939 | 0.061 |
| 3 | 1.970 | 0.030 | 1.829 | 0.171 | 1.769 | 0.231 | 1.922 | 0.078 | 1.928 | 0.072 |
| 4 | 1.825 | 0.175 | 1.847 | 0.153 | 1.880 | 0.120 | 1.849 | 0.151 | 1.929 | 0.071 |
| 5 | 1.812 | 0.188 | 1.909 | 0.091 | 1.823 | 0.177 | 1.915 | 0.085 | 1.924 | 0.076 |
| 6 | 1.862 | 0.138 | 1.833 | 0.167 | 1.791 | 0.209 | 1.915 | 0.085 | 1.896 | 0.104 |
| 7 | 1.989 | 0.011 | 1.920 | 0.080 | 1.836 | 0.164 | 1.896 | 0.104 | 1.907 | 0.093 |
| 8 | 1.801 | 0.199 | 1.997 | 0.003 | 1.799 | 0.201 | 1.958 | 0.042 | 1.904 | 0.096 |
| 9 | 1.954 | 0.046 | 1.882 | 0.118 | 1.857 | 0.143 | 1.760 | 0.240 | 1.916 | 0.084 |
| 10 | | | | | | | | | 1.868 | 0.132 |
| 11 | | | | | | | | | 1.913 | 0.087 |
| 12 | | | | | | | | | 1.950 | 0.050 |
| | | | | | | | | | 1.952 | 0.048 |
| Fluorinated | | | | | | | | | | |
| VBM-N | (0001) | | (111) | | (110) | | $(10\bar{1}0)$ | | $(\bar{2}110)$ | |
| | C | F | C | F | C | F | C | F | C | F |
| N=0 | 0.919 | 1.081 | 1.091 | 0.909 | 0.972 | 1.028 | 0.985 | 1.015 | 1.241 | 0.759 |
| N=1 | 0.833 | 1.167 | 0.857 | 1.143 | 0.763 | 1.237 | 0.869 | 1.131 | 1.129 | 0.871 |
| 2 | 1.355 | 0.645 | 1.388 | 0.612 | 0.879 | 1.121 | 1.197 | 0.803 | 0.997 | 1.003 |
| 3 | 1.133 | 0.867 | 1.206 | 0.794 | 0.821 | 1.179 | 1.195 | 0.805 | 1.044 | 0.956 |
| 4 | 1.566 | 0.434 | 1.123 | 0.877 | 1.091 | 0.909 | 1.071 | 0.929 | 1.021 | 0.979 |
| 5 | 1.514 | 0.486 | 1.563 | 0.437 | 0.794 | 1.206 | 0.966 | 1.034 | 0.867 | 1.133 |
| 6 | 1.619 | 0.381 | 1.464 | 0.536 | 1.052 | 0.948 | 1.512 | 0.488 | 0.952 | 1.048 |
| 7 | 1.536 | 0.464 | 1.638 | 0.362 | 0.921 | 1.079 | 1.102 | 0.898 | 0.818 | 1.182 |

| | | | | | | | | | | |
|---|---|---|---|---|---|---|---|---|---|---|
| 8 | 1.564 | 0.436 | 1.471 | 0.529 | 0.914 | 1.086 | 1.299 | 0.701 | 1.222 | 0.778 |
| 9 | 1.450 | 0.550 | 1.625 | 0.375 | 1.004 | 0.996 | 1.443 | 0.557 | 1.243 | 0.757 |
| 10 | 1.479 | 0.521 | 1.629 | 0.371 | 1.354 | 0.646 | 1.540 | 0.460 | 1.203 | 0.797 |
| 11 | 1.650 | 0.350 | 1.403 | 0.597 | 1.585 | 0.415 | 1.344 | 0.656 | 1.048 | 0.952 |
| 12 | 1.551 | 0.449 | 1.724 | 0.276 | 1.558 | 0.442 | 1.555 | 0.445 | 1.052 | 0.948 |
| 13 | 1.547 | 0.453 | 1.620 | 0.380 | 1.613 | 0.387 | 1.561 | 0.439 | 1.193 | 0.807 |
| 14 | 1.695 | 0.305 | 1.392 | 0.608 | 1.511 | 0.489 | 1.638 | 0.362 | 1.038 | 0.962 |
| 15 | 1.550 | 0.450 | 1.723 | 0.277 | 1.516 | 0.484 | 1.760 | 0.240 | 1.149 | 0.851 |
| 16 | 1.654 | 0.346 | 1.580 | 0.420 | 1.181 | 0.819 | 1.654 | 0.346 | 1.281 | 0.719 |
| 17 | 1.619 | 0.381 | 1.770 | 0.230 | 1.186 | 0.814 | 1.677 | 0.323 | 1.072 | 0.928 |
| 18 | 1.756 | 0.244 | 1.787 | 0.213 | 1.178 | 0.822 | 1.673 | 0.327 | 1.486 | 0.514 |
| 19 | 1.714 | 0.286 | 1.628 | 0.372 | 1.264 | 0.736 | 1.633 | 0.367 | 1.489 | 0.511 |
| 20 | | | | | | | 1.658 | 0.342 | 1.538 | 0.462 |
| 21 | | | | | | | 1.488 | 0.512 | 1.448 | 0.552 |
| 22 | | | | | | | 1.520 | 0.480 | 1.645 | 0.355 |
| 23 | | | | | | | 1.452 | 0.548 | 1.651 | 0.349 |
| 24 | | | | | | | 1.469 | 0.531 | 1.580 | 0.420 |
| 25 | | | | | | | 1.542 | 0.458 | 1.540 | 0.460 |
| 26 | | | | | | | 1.606 | 0.394 | 1.735 | 0.265 |
| 27 | | | | | | | 1.384 | 0.616 | 1.750 | 0.250 |
| 28 | | | | | | | 1.462 | 0.538 | 1.542 | 0.458 |
| 29 | | | | | | | 1.466 | 0.534 | 1.543 | 0.457 |
| 30 | | | | | | | | | 1.419 | 0.581 |
| 31 | | | | | | | | | 1.602 | 0.398 |
| 32 | | | | | | | | | 1.646 | 0.354 |
| 33 | | | | | | | | | 1.710 | 0.290 |
| 34 | | | | | | | | | 1.752 | 0.248 |
| 35 | | | | | | | | | 1.721 | 0.279 |
| 36 | | | | | | | | | 1.649 | 0.351 |
| 37 | | | | | | | | | 1.778 | 0.222 |
| 38 | | | | | | | | | 1.695 | 0.305 |
| 39 | | | | | | | | | 1.643 | 0.357 |
| 40 | | | | | | | | | 1.702 | 0.298 |
| 41 | | | | | | | | | 1.729 | 0.271 |
| 42 | | | | | | | | | 1.643 | 0.357 |
| 43 | | | | | | | | | 1.668 | 0.332 |
| 44 | | | | | | | | | 1.555 | 0.445 |
| 45 | | | | | | | | | 1.656 | 0.344 |
| 46 | | | | | | | | | 1.501 | 0.499 |
